# Longitudinal fecal microbiome and metabolite data demonstrate rapid shifts and subsequent stabilization after an abrupt dietary change in healthy adult dogs

**DOI:** 10.1186/s42523-022-00194-9

**Published:** 2022-08-01

**Authors:** Ching-Yen Lin, Aashish R. Jha, Patrícia M. Oba, Sofia M. Yotis, Justin Shmalberg, Ryan W. Honaker, Kelly S. Swanson

**Affiliations:** 1 Division of Nutritional Sciences, University of Illinois at Urbana-Champaign, Urbana, IL 61801 USA; 2 NomNomNow, Inc, Nashville, TN 37218 USA; 3 Genetic Heritage Group, Department of Biology, New York University Abu Dhabi, Abu Dhabi, UAE; 4 Department of Animal Sciences, University of Illinois at Urbana-Champaign, Urbana, IL 61801 USA; 5 Department of Comparative, Diagnostic and Population Medicine, College of Veterinary Medicine, University of Florida, Gainesville, FL 32608 USA; 6 Department of Veterinary Clinical Medicine, University of Illinois at Urbana-Champaign, Urbana, IL 61801 USA

**Keywords:** Canine metagenome, Gastrointestinal health, Gut microbiota, Fecal metabolites, Microbial function

## Abstract

**Background:**

Diet has a large influence on gut microbiota diversity and function. Although previous studies have investigated the effect of dietary interventions on the gut microbiome, longitudinal changes in the gut microbiome, microbial functions, and metabolite profiles post dietary interventions have been underexplored. How long these outcomes require to reach a steady-state, how they relate to one another, and their impact on host physiological changes are largely unknown. To address these unknowns, we collected longitudinal fecal samples following an abrupt dietary change in healthy adult beagles (n = 12, age: 5.16 ± 0.87 year, BW: 13.37 ± 0.68 kg) using a crossover design. All dogs were fed a kibble diet (control) from d1-14, and then fed that same diet supplemented with fiber (HFD) or a protein-rich canned diet (CD) from d15-27. Fresh fecal samples were collected on d13, 16, 20, 24, and 27 for metabolite and microbiome assessment. Fecal microbial diversity and composition, metabolite profiles, and microbial functions dramatically diverged and stabilized within a few days (2 d for metabolites; 6 d for microbiota) after dietary interventions. Fecal acetate, propionate, and total short-chain fatty acids increased after change to HFD, while fecal isobutyrate, isovalerate, total branched-chain fatty acids, phenol, and indole increased after dogs consumed CD. Relative abundance of ~ 100 bacterial species mainly belonging to the Firmicutes, Proteobacteria, and Actinobacteria phyla increased in HFD. These shifts in gut microbiome diversity and composition were accompanied by functional changes. Transition to HFD led to increases in the relative abundance of KEGG orthology (KO) terms related to starch and sucrose metabolism, fatty acid biosynthesis, and amino sugar and nucleotide sugar metabolism, while transition to CD resulted in increased relative abundance of KO terms pertaining to inositol phosphate metabolism and sulfur metabolism. Significant associations among fecal microbial taxa, KO terms, and metabolites were observed, allowing for high-accuracy prediction of diet group by random forest analysis.

**Conclusions:**

Longitudinal sampling and a multi-modal approach to characterizing the gastrointestinal environment allowed us to demonstrate how drastically and quickly dietary changes impact the fecal microbiome and metabolite profiles of dogs following an abrupt dietary change and identify key microbe-metabolite relationships that allowed for treatment prediction.

**Supplementary Information:**

The online version contains supplementary material available at 10.1186/s42523-022-00194-9.

## Background

Dietary macronutrient content is one of the key factors shaping the composition, metabolic activity, and diversity of the fecal microbiome and concentrations of fecal metabolites [1, 2]. Dietary fibers and other non-digestible carbohydrates have been the most studied and are well known for their positive influence on gastrointestinal microbiota populations and activity [3–8]. Dietary fibers have the potential to increase intestinal production of short-chain fatty acids (SCFA) and lactate, increasing microbial metabolic activity and enrichment of bacteria in the Firmicutes phylum and other saccharolytic taxa [9–11]. SCFA contribute to normal bowel function and prevent pathology. Likewise, the amount and type of dietary protein and/or fat may also affect the composition of fecal microbiota [12–17]. High-protein diets can increase putrefactive metabolites (ammonia, indoles, amines) due to amino acid degradation by the intestinal microbiota, and increase Fusobacteria and Proteobacteria abundance [18]. High-fat diets can increase fecal bile acid concentrations and have a negative impact on Prevotella and xylan fermentation [19].

The overall impact that diet has on the gastrointestinal microbiota has been recently reported in canines. The kinetics required by a dietary change to modify the community structure and activity of gut microbiota and then stabilize, however, has not been well studied. Most dietary intervention studies provide a dietary treatment for 2 to 4 weeks prior to sample collection to allow the microbial community and activity to stabilize [20]. A limited number of studies have measured short-term microbial dynamics in humans [21–23], but with low subject numbers and a lack of longitudinal sampling times. The best attempt to characterize these short-term microbial changes following a diet change was that reported by David et al. [21], who demonstrated that a shift to diets composed entirely of animal or plant products rapidly altered microbial community structure and gene expression in adult humans. Despite the interesting findings, fecal microbiota and metabolites were only measured over a short period of time (i.e., 5-day diet intervention) that did not allow for possible stabilization.

Microbial community metabolomics is a new approach to identify functional differences in the microbiome. It is capable of identifying a large number of small-molecule metabolites in microbial communities [24]. Within metabolomics, nutritional metabolomics aims to clarify the functional responses between the effects of different diets [25–27]. The analysis of metabolic differences between biochemical pathways can help discover potential biomarkers associated with deleterious effects to the host and provide tools for a better understanding of the pathogenesis of diseases, and possibly how diets can help prevent or aid in disease therapy [25–30]. To use this information in practice, a better understanding of how dietary changes may impact microbial communities and their ability to produce bioactive molecules is needed.

Although many studies have investigated how dietary interventions affect gut communities after a given treatment, longitudinal changes in gut microbial composition, microbial function, and metabolite profiles soon after a dietary change have been underexplored. The necessary time required for these outcomes to reach a steady state, how they relate to one another, and their impact on host physiological changes are still largely unknown. For those reasons, the primary objective of this study was to determine the timing and shifts of fecal microbiota and metabolites of dogs shifted to vastly different diets (high-fiber extruded diet; high-protein, high-fat canned diet). Our secondary objective was to identify microbe-KEGG orthology (KO) term-metabolite relationships and prediction of dietary intervention groups by random forest.

## Results

### Fecal characteristics and metabolites rapidly shift with diet transition

**Fig. 1:**
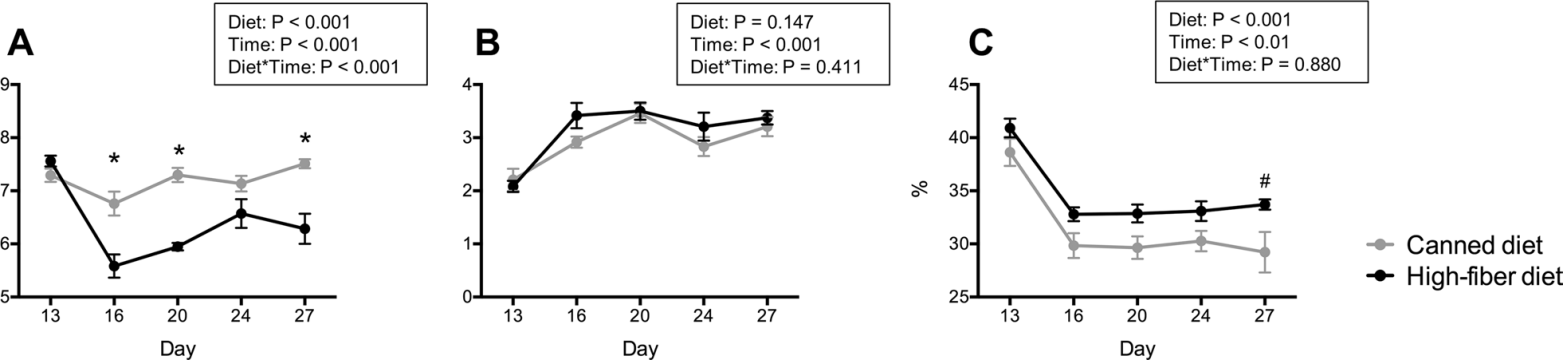
Fecal characteristics, including fecal pH (**A**), fecal scores (**B**), and fecal dry matter (**C**) of dogs fed a high-fiber diet or protein-rich canned diet. *Mean values within time points that were different between diets (*P* < 0.05); #Mean values within time points that tended to be different between diets (P < 0.10). Fecal samples were scored according to a 5-point scale: 1 = hard, dry pellets, small hard mass; 2 = hard, formed, dry stool; remains firm and soft; 3 = soft, formed, and moist stool, retains shape; 4 = soft, unformed stool, assumes shape of container; and 5 = watery, liquid that can be poured

After diet transition, fecal pH was affected by diet and time (Fig. 1A). Dogs transitioned to a high-fiber diet (HFD) had a rapid reduction in fecal pH, which was lower (*P* < 0.001) than that of dogs fed a canned diet (CD) at most time points (d 16, d 20, and d 24). Dogs transitioned to CD had a slight increase in fecal pH over time. Fecal score rapidly increased (*P* < 0.001; looser stools) in dogs transitioned to both diets (Fig. 1B). Fecal dry matter (DM) was affected by diet and time (Fig. 1C). Fecal DM was rapidly reduced after diet transition and remained lower than baseline over time. Even though both groups were affected by diet change, dogs transitioned to HFD had a greater (*P* < 0.001) fecal DM compared with those fed CD.

**Fig. 2:**
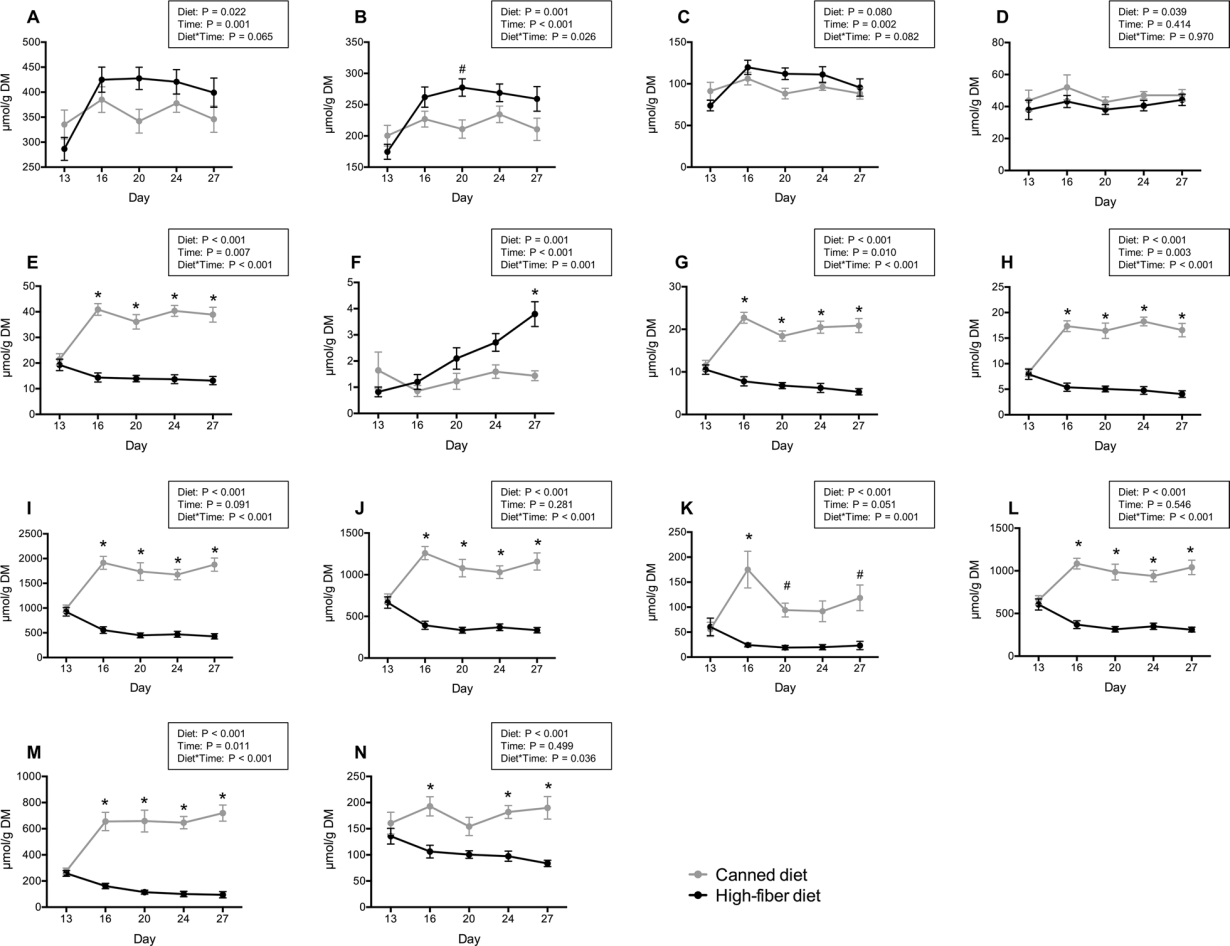
Fecal total short-chain fatty acids (**A**), acetate (**B**), propionate (**C**), butyrate (**D**), total branched-chain fatty acids (**E**), valerate (**F**), isovalerate (**G**), isobutyrate (**H**), total phenol and indole (**I**), total phenol (**J**), phenol (**K**), 4-ethylphenol (**L**), indole (**M**), and ammonia (**N**) of dogs fed a high-fiber diet or protein-rich canned diet. *Mean values within time points that were different between diets (*P* < 0.05); ^#^Mean values within time points that tended to be different between diets (*P* < 0.10)

**Fig. 3:**
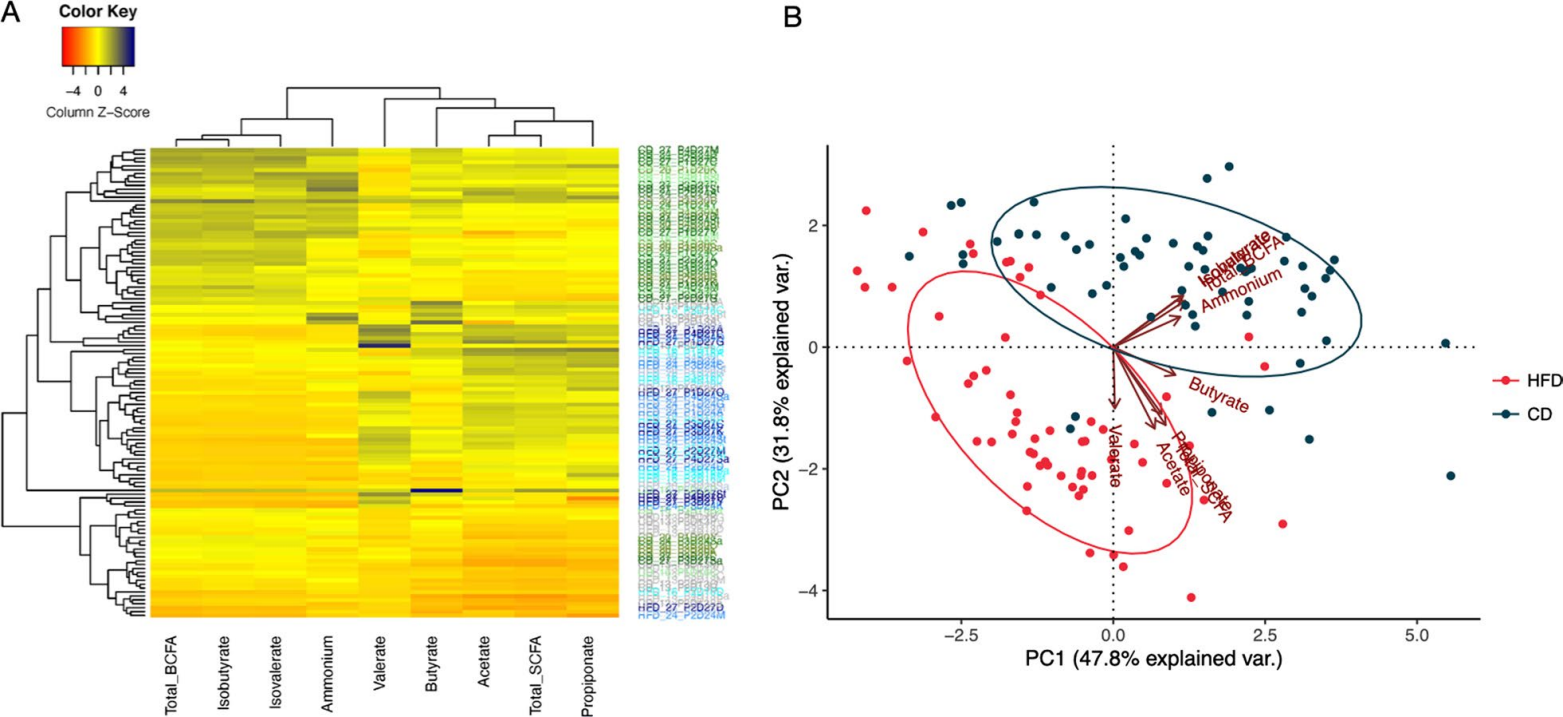
**A** Heatmap showing differences in fecal metabolites including short-chain fatty acids (SCFA), branched-chain fatty acids (BCFA) and ammonia in different diet groups [grey: control diet at baseline (d 13); green: high-fiber diet (HFD); blue: protein-rich canned diet (CD)] **B** PCA plot showing fecal metabolites of the HFD or CD treatment groups clustering together and separately

Fecal metabolites were rapidly and strongly affected by both diet and time following diet transition. Fecal total SCFA, acetate, and propionate concentrations increased in dogs fed HFD and were higher than those fed CD (Fig. 2 A–C). Fecal butyrate concentrations were not affected by time, but were greater (*P* < 0.05) in dogs transitioned to CD (Fig. 2D). Fecal total branched-chain fatty acids (BCFA), isovalerate, isobutyrate, total phenol and indole, phenol, 4-ethylphenol, indole, and ammonia concentrations rapidly increased following transition to CD and were higher (*P* < 0.001) in dogs fed CD compared with HFD (Fig. 2E and G–N). Fecal valerate was impacted by diet and time, with concentrations being relatively stable in dogs transitioned to CD, but increasing over time in dogs transitioned to HFD (Fig. 2F). The heatmap presented in Fig. 3A shows how dogs clustered by diet (HFD-green, CD-blue, and baseline-grey) based on fecal metabolite profiles. The PCA plot in Fig. 3B shows the relationship of fecal metabolite profiles of dogs fed HFD and CD. Dietary treatments clustered together and away from each other, with the HFD being most closely associated with acetate, propionate, total SCFA, and valerate and CD most closely associated with isobutyrate, ammonia, and total BCFA.

### Bacterial alpha and beta diversity rapidly shift with diet transition

**Fig. 4:**
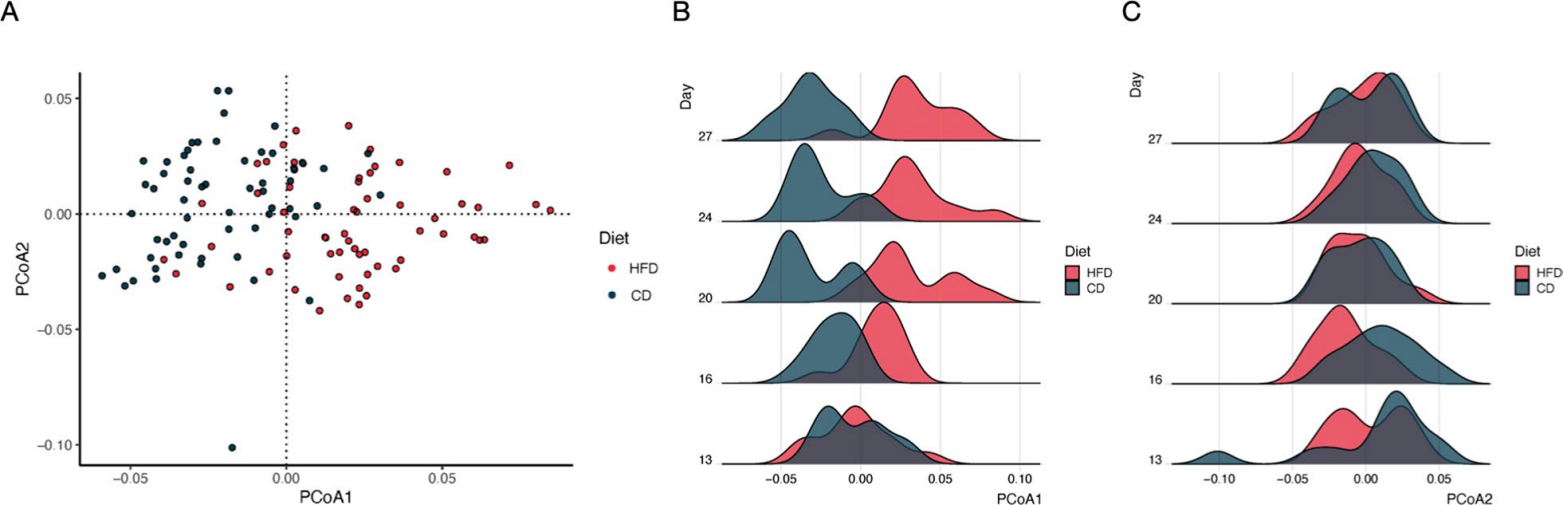
Shifts in fecal microbiome composition between the high-fiber diet (HFD) and the protein-rich canned diet (CD) groups over time from shotgun sequencing. **A** Principal Coordinate Analysis (PCoA) of species-level fecal microbiomes from all time points using Jensen-Shannon distance. **B** The fecal bacterial composition was comparable between the diet groups on d 13 when the dietary regimen started. Microbiome composition shifted in opposite directions along PCoA1 over time between diet groups. **C** No significant shift was observed along PCoA2 over time. PERMANOVA was used to assess the association between diet and period with the fecal microbiome composition. Kruskal-Wallis test was used to assess association between diets and the top two axes (PCo1, PCo2) followed by Dunn’s post-hoc test to evaluate the difference over time within each dietary group

A total of 84.3 million reads (average 1.4 million reads/sample) from shotgun sequencing were aligned to a bacterial database after quality control and filtering of low-quality reads. Principal coordinates analysis (PCoA) using Jensen-Shannon distance (Fig. 4A) at the species level revealed distinct separations of HFD and CD groups (*P* < 0.001). Additionally, the bacterial composition of the diet groups shifted in opposite directions (*P* < 0.05) over time along the 1st principal coordinate, but not along the 2nd principal coordinate after diet transition (Fig. 4B and C). Data from 16S rRNA gene sequencing (even sampling depth of 24,423 sequences) also showed rapid shifts in alpha and beta diversity measures (Additional file 1: Fig. S1), with separate clusters present by d 16 (weighted) and d 20 (unweighted).

### Random forest analysis of shotgun data predicts dietary shifts in bacterial taxonomy, enzymes, and modules

**Fig. 5:**
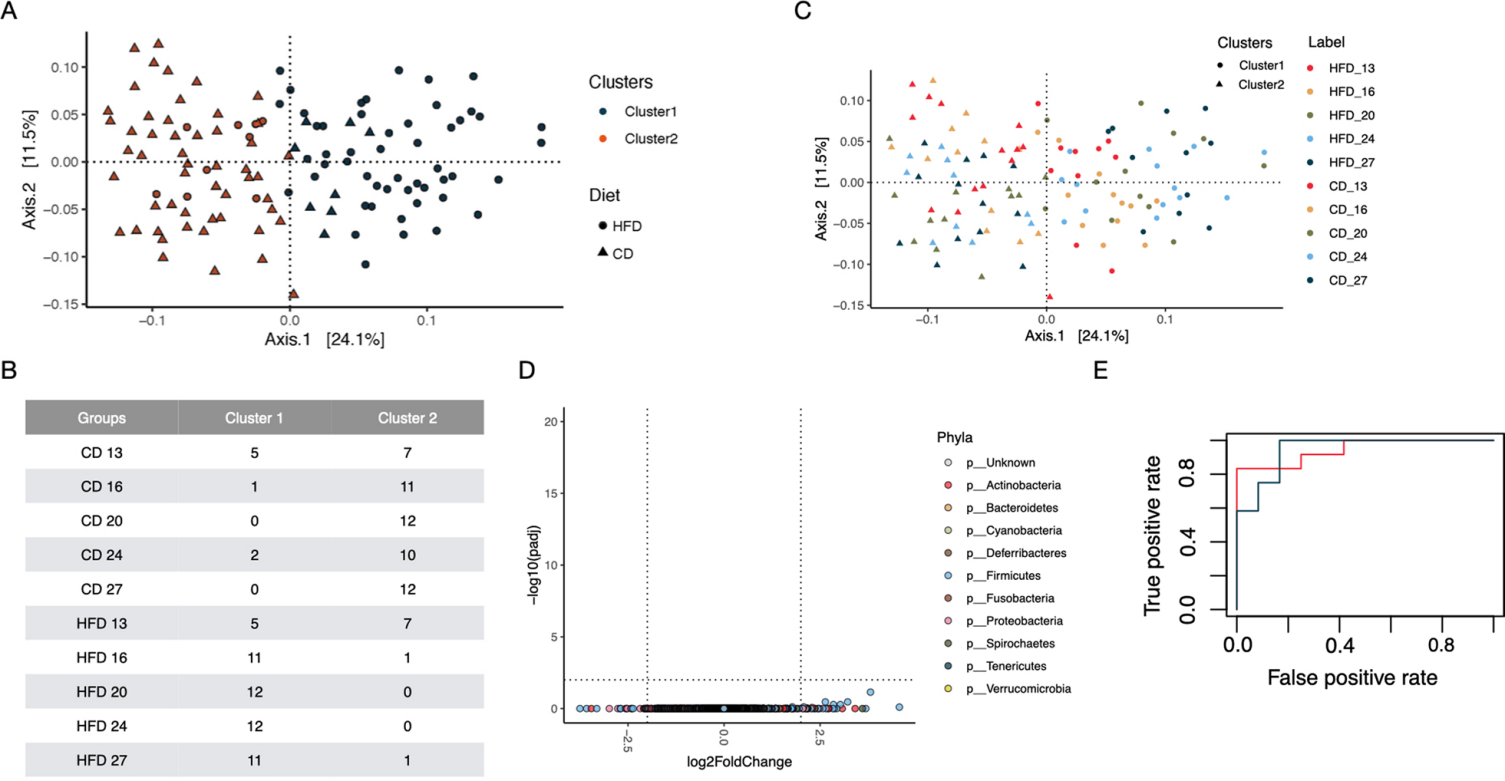
Differences between groups and between time points within each group. **A** Partitioning around medoids clustering of fecal microbiota using the top two PCoA axes (Fig. 4) revealed dogs in this study can be partitioned into two clusters (blue and red). **B**–**C** Cluster 1 contained primarily time points from the high-fiber diet (HFD) and Cluster 2 was enriched with time points from the protein-rich canned diet (CD). At the start of the trial (i.e., d 13), however, individuals were almost evenly split between the two clusters. This agrees with DESeq that showed no differences in genus abundance between the two diet groups at d 13 (**D**). **E** Moreover, a random forest (RF) classifier accurately differentiated the microbial compositions by diet

Before the diet change (i.e., d 13), dogs were almost evenly split between the two clusters, indicating that their microbiomes were similar. No differences in genus abundance between the two diet groups were observed at d 13 (*P* > 0.05). Random forest analysis, however, accurately differentiated the microbial compositions by diet following diet transition. Based on the microbiome data alone, individuals could be grouped into two clusters (Fig. 5). Cluster 1 contained primarily time points from HFD and Cluster 2 was enriched for time points from CD.

### Bacterial taxonomy affected by diet

**Fig. 6:**
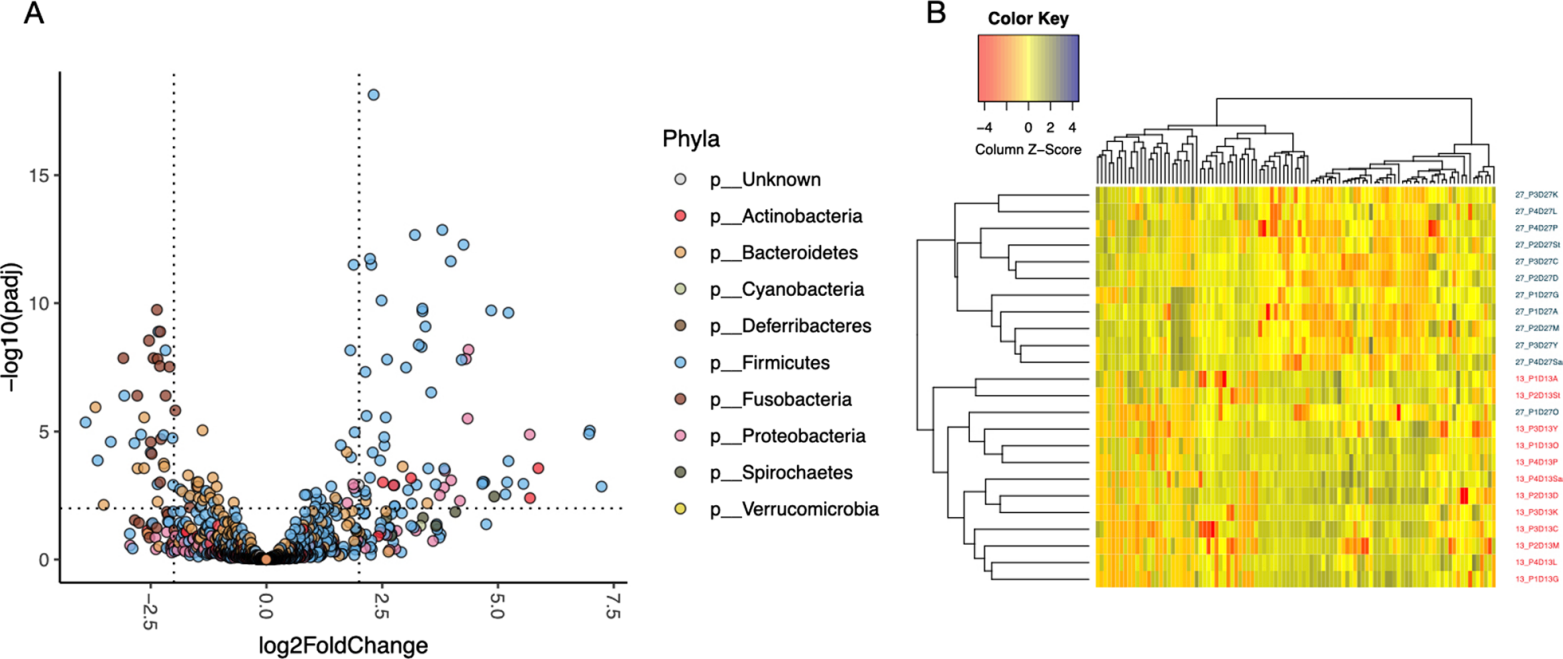
Bacterial species associated with the high-fiber diet. **A** Volcano plot showing differential abundance of bacterial species in the high-fiber diet group between d 13 vs. d 27. Each dot is a bacterial species and dots are colored by phylum. Positive values in x-axis represent species that had higher relative abundance after a high-fiber diet was administered for 14 d (d 27) relative to baseline. Negative values in the x-axis represent species that decreased in abundance in the high-fiber diet group relative to the baseline. The horizontal dotted line represents significance threshold of FDR adjusted *P* < 0.01 obtained from DESeq2 and the two vertical lines differentiate the species with log2 fold change in abundance. Species with FDR adjusted *P* < 0.01 and absolute log2 fold change > 2 were considered statistically significant. **B** Heatmap showing differences in relative abundance of differentially abundant bacterial species between d 13 (red) and d 27 (green)

Shotgun sequencing demonstrated that while the relative abundances of bacterial species were not different between diet groups at baseline (i.e., d 13), the relative abundances of 99 bacterial species were increased (*P* < 0.01) and 59 species were decreased (*P* < 0.01) by d 27 in dogs fed HFD (Fig. 6A). For example, the relative abundances of several species in the *Bifidobacterium*, *Brachyspira*, *Clostridium*, *Helicobacter*, *Lactobacillus*, and *Megamonas* genera, many key species of well-known fiber degraders and SCFA/butyrate producers (*Dorea*; *Roseburia*; *Blautia*; *Prevotella*), and a few species in the *Eubacterium*, *Lachnoclostridium*, *Massilioclostridum*, and *Streptococcus* genera were enriched in animals fed HFD compared with baseline. Additionally, the relative abundances of several species in the *Bacteroides*, *Cetobacterium*, and *Clostridium*, and *Fusobacterium* genera and two species in the *Coprococcus* genera were reduced in animals fed HFD compared with baseline. The bacterial species compositions between baseline and d 27 of HFD-fed dogs were distinct, as shown in the heatmap in Fig. 6B.

Compared with baseline, the relative abundances of four *Megamonas* spp., two *Lactobacillus* spp., and several other taxa within the Firmicutes phyla were increased in animals fed HFD at all-time points (16, 20, 24, and 27) (Additional file 1: Table S1). Compared with baseline, the relative abundance of *Alistipes* spp. was increased and *Campylobacter fetus* were decreased on d 16 and 20 of dogs fed HFD (Additional file 1: Table S2) while the relative abundance of *Intestinimonas butyriciproducens* was decreased on d 16, 20, and 24 of dogs fed HFD (Additional file 1: Table S2). Compared with baseline, the relative abundances of three *Fusobacterium* spp., four species of *Clostridium*, *Bacillus bogoriensis*, *Bacteroides paurosaccharolyticus*, unclassified *Butyrivibrio*, and unclassified *Turicibacter* were increased, while several *Clostridium, Anaerostipes hadrus*, *Bacteroides* spp., *Erysipelotrichaceae bacterium*, *Faecalitalea cylindroides*, *Lachnospira multipara*, *Lachnospiraceae bacterium*, *Massiliomicrobiota timonensis*, *Nostoc linckia*, *Prevotella bergensis*, *Streptomyces sampsonii*, and unclassified Campylobacterales were decreased on d 24 and 27 of dogs fed HFD (Additional file 1: Table S2). Compared with baseline, the relative abundances of several *Bifidobacterium* and *Lactobacillus* species and *Blautia*, *Catonella morbi*, *Coprobacillus*, *Dialister succinatiphilus*, *Dubosiella newyorkensis*, *Erysipelotrichaceae bacterium*, *Gemmiger formicilis*, *Helicobacter cinaedi*, *Holdemanella biformis*, *Lachnospiraceae bacterium*, *Massilioclostridium coli*, *Megasphaera elsdenii*, *Porphyromonas somerae*, *Sharpea azabuensis*, *Solobacterium moorei*, and *Streptococcus* spp. were increased, while the relative abundances of *Bacteroides* spp. HPS0048, *Bacteroides cellulosilyticus*, *Petrimonas mucosa*, *Enterococcu*s spp., and *Ilyobacter polytropus* were decreased on d 20, 24, and 27 of dogs fed HFD (Additional file 1: Table S3).

**Fig. 7:**
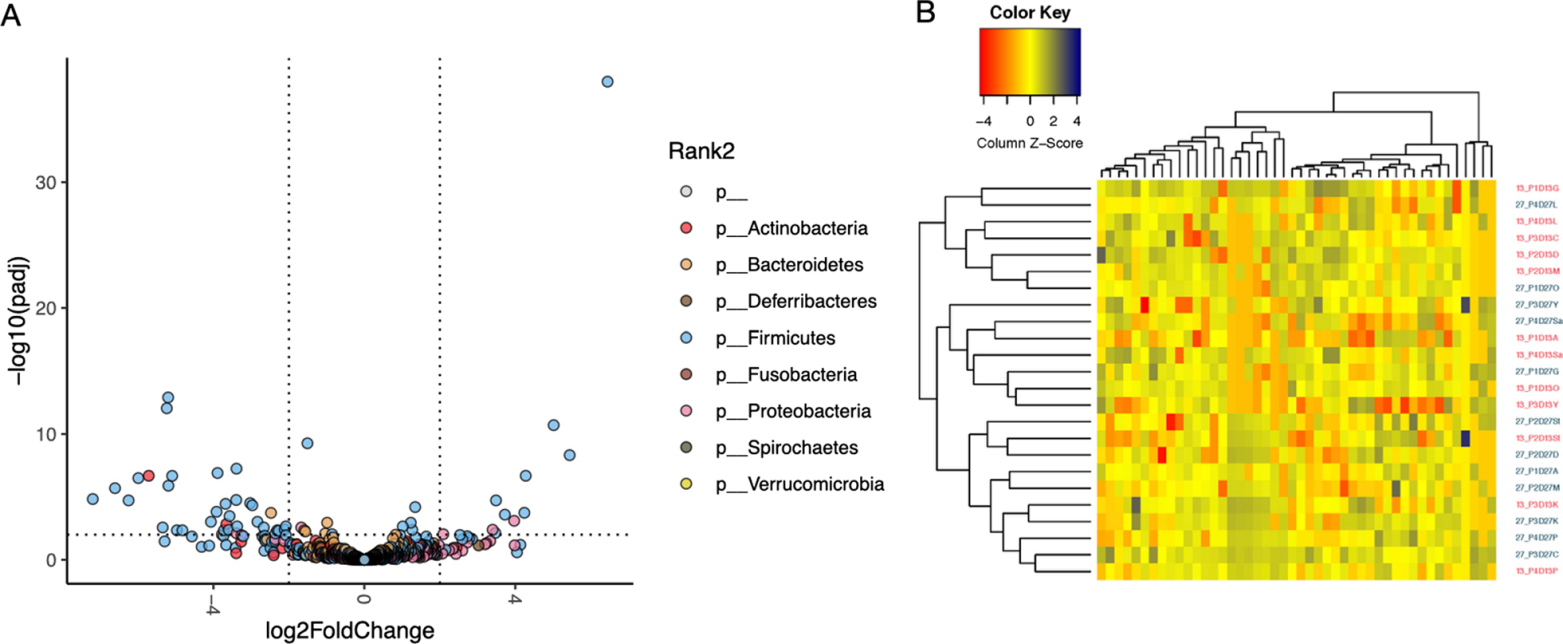
Bacterial species associated with the protein-rich canned diet. **A** Volcano plot showing differential abundance of bacterial species in the protein-rich canned diet group between d 13 vs. d 27. Each dot is a bacterial species and dots are colored by phylum. Positive values in x-axis represent species that had higher relative abundance after fiber diet was administered for 14 d (d 27) relative to baseline. Negative values in the x-axis represent species that decreased in abundance in the protein-rich canned diet relative to the baseline. The horizontal dotted line represents significance threshold of FDR adjusted *P* < 0.01 obtained from DESeq2 and the two vertical lines differentiate the species with log2 fold change in abundance. Species with FDR adjusted *P* < 0.01 and absolute log2 fold change > 2 were considered statistically significant. **B** Heatmap showing differences in relative abundance of differentially abundant bacterial species between d 13 (red) and d 27 (green)

Compared with baseline, the relative abundances of 22 bacterial species were increased (*P* < 0.01), while the relative abundances of 56 bacterial species were decreased (P < 0.01) by d 27 of dogs fed CD (Fig. 7A). In these dogs, the relative abundances of several *Citrobacter* and *Lactococcu*s species were enriched compared with baseline. Also, the relative abundances of several species of *Lactobacillus* and *Streptococcus*, and a few species of *Bifidobacterium*, *Enterococcus*, *Erysipelatoclostridium, Faecalibaculum*, *Megamonas*, and *Turicibacter* were reduced in animals fed CD compared with baseline. The species compositions between baseline and d 27 of CD-fed dogs were not as distinct as those fed HFD, with no clear separation of the two time points observed in the heatmap (Fig. 7B).

Compared with baseline, the relative abundances of *Candidatus Dorea massiliensis*, *Stomatobaculum longum*, and *Paeniclostridium sordellii* were enriched, while the relative abundances of several *Streptococcus* spp., two *Bifidobacterium* spp., two *Clostridium* spp., and one unclassified Enterococcaceae spp. were reduced in animals fed CD at all time points (16, 20, 24, and 27) (Additional file 1:  Table S4). Compared with baseline, the relative abundance of *Streptococcus parasanguinis* was reduced on d 16, 20, and 24 of dogs fed CD (Additional file 1: Table S5). Compared with baseline, the relative abundances of *Lactococcus lactis, Niameybacter massiliensis, Terrisporobacter glycolicus*, and unclassified *Citrobacter* were enriched, while the relative abundances of *Olsenella* spp., *Enterococcus columbae*, *Lactobacillus acidophilus*, *Streptococcus mitis*, and *Fournierella massiliensis* were reduced on d 24 and 27 of dogs fed CD (Additional file 1: Table S5). Compared with baseline, the relative abundances of *Enterococcus* spp., two *Lactococcus* spp., *Clostridium paraputrificum*, *Paraclostridium bifermentans*, *Enterobacter mori*, and unclassified *Lactococcus* were enriched, while the relative abundances of several *Lactobacillus*, two species of *Streptococcus* and *Enterococcus*, and *Megamonas*, *Parabacteroides* spp., *Atopostipes suicloacalis*, *Romboutsia timonensis*, *Faecalibaculum rodentium*, *Turicibacter* spp., *Burkholderiales bacterium*, *Parasutterella excrementihominis*, *Campylobacter fetus*, unclassified Lactobacillaceae, unclassified *Turicibacter*, and unclassified Burkholderiales were reduced on d 20, 24, and 27 of dogs fed CD (Additional file 1: Table S6). 16S rRNA gene sequencing data are reported in Additional file 1: Table S7.

### Bacterial gene (KO term) abundance and functional modules affected by diet

**Fig. 8:**
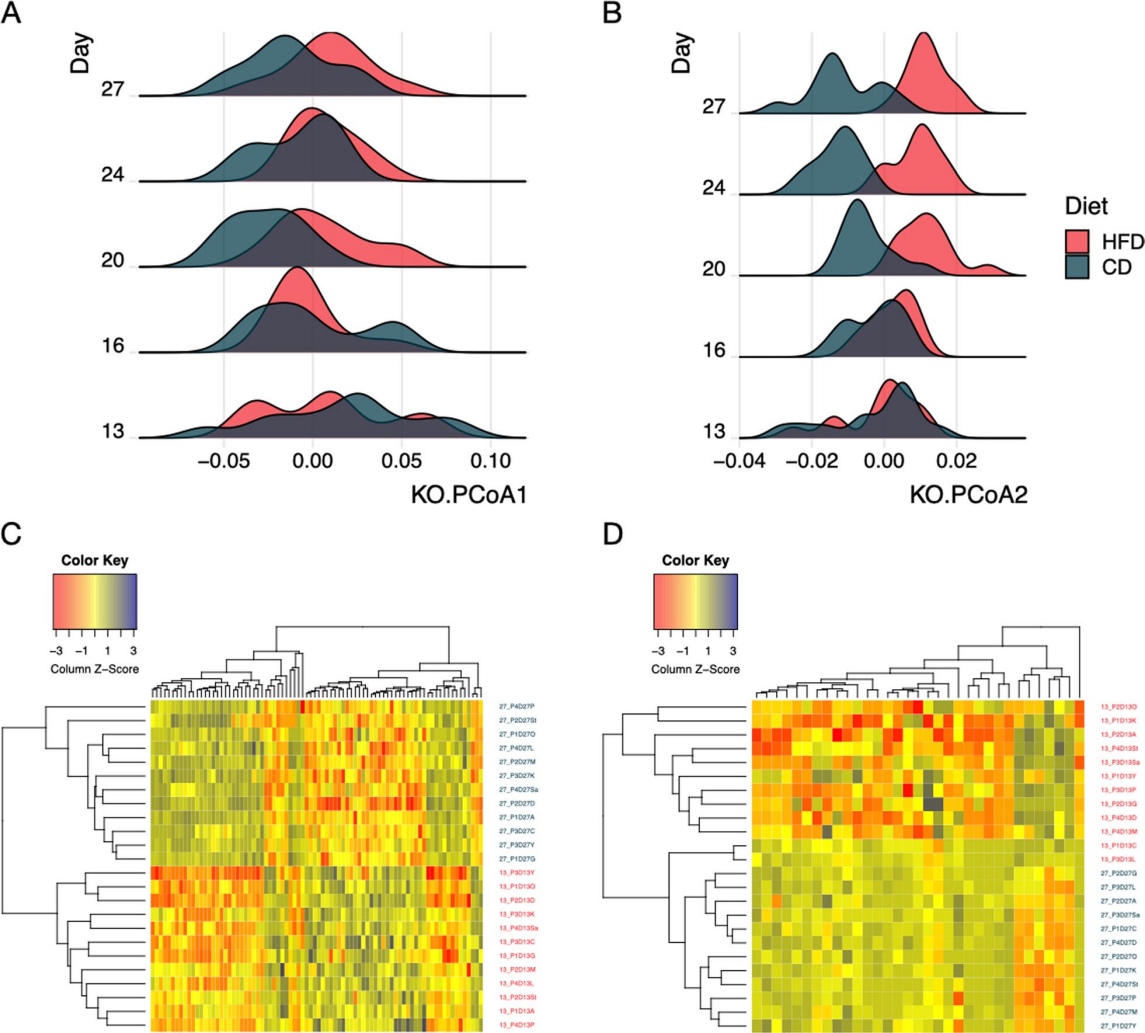
Shifts in predicted gut microbial functions between the high-fiber diet (HFD) and the protein-rich canned diet (CD) groups over time. **A** Principal Coordinate Analysis (PCoA) of KO using Jensen-Shannon distance shows that KO terms were comparable between the diet groups on d 13 when the dietary regimen started. No significant shift was observed along PCoA1. **B** KO terms shifted in opposite directions along PCoA2 over time between diet groups. A Kruskal-Wallis test was used to assess association between diets and the top two PCoA axes followed by Dunn’s post-hoc test to evaluate the difference between groups. Heatmaps show differences in relative abundance of KO terms between d 13 (red) and d 27 (green) in the HFD group (**C**) and the CD group (**D**)

PCoA was used to predict shifts in gut microbial functionality in dogs fed HFD or CD (Fig. 8). The figure demonstrates that KO terms were comparable between the diet groups on d 13 prior to dietary change. No significant shift was observed along the 1st principal coordinate after the dietary shift (Fig. 8A). However, KO terms shifted in opposite directions along the 2nd principal coordinate over time between the diet groups, with a clear separation observed between dietary groups by d 20 (Fig. 8B). Heatmaps based on KO term relative abundances show a clear separation between d 13 (red) and d 27 (green) of dogs fed HFD (Fig. 8C) and those fed CD (Fig. 8D).

The metabolic super-pathways positively affected at all time points after diet transition (on d 16, 20, 24, and 27) of dogs fed HFD were those associated with carbohydrate metabolism, lipid metabolism, xenobiotic biodegradation and metabolism, amino acid metabolism, metabolism of terpenoids and polyketides, biosynthesis of other secondary metabolites, metabolism of cofactors and vitamins, and amino acid metabolism (Additional file 1: Table S8). The metabolic super-pathways positively affected on d 20, 24, and 27 of dogs fed HFD were those associated with the metabolism of cofactors and vitamins, amino acid metabolism, metabolism of terpenoids and polyketides, and carbohydrate metabolism (Additional file 1: Table S8). The metabolic super-pathways positively affected only on d 24 and 27 of dogs fed HFD were those associated with amino acid metabolism, carbohydrate metabolism, and energy metabolism (Additional file 1: Table S8).

The metabolic super-pathway less represented on d 16, 24, and 27 of dogs fed HFD was associated with xenobiotic biodegradation and metabolism (Additional file 1: Table S9). The metabolic super-pathways negatively affected on d 20, 24, and 27 of dogs fed HFD were those associated with carbohydrate metabolism, xenobiotic biodegradation and metabolism, and metabolism of other amino acids (Additional file 1: Table S9). The metabolic super-pathways negatively affected only on d 24 and 27 of dogs fed HFD were those associated with energy metabolism, lipid metabolism, glycan biosynthesis and metabolism, and biosynthesis of other secondary metabolites (Additional file 1: Table S9).

The metabolic super-pathways positively affected on d 20, 24, and 27 of dogs fed CD were associated with energy metabolism, carbohydrate metabolism, and amino acid metabolism (Additional file 1: Table S10). The primary metabolic super-pathway that was negatively affected on d 20, 24, and 27 of dogs fed CD was associated with carbohydrate metabolism (Additional file 1: Table S10). The metabolic super-pathways positively affected only on d 24 and 27 of dogs fed by CD were those associated with carbohydrate metabolism, lipid metabolism, metabolism of terpenoids and polyketides, xenobiotic biodegradation and metabolism, energy metabolism, amino acid metabolism, and nucleotide metabolism (Additional file 1: Table S10).

### Microbiota-metabolite relationships

**Fig. 9:**
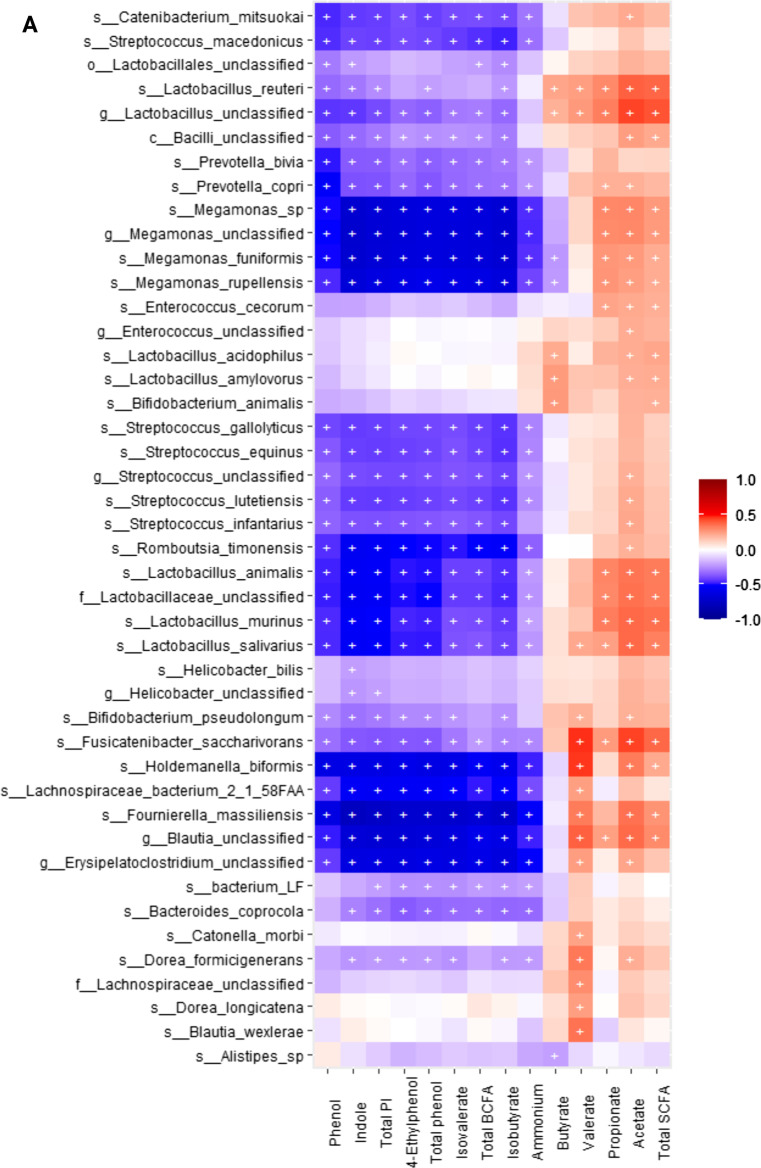

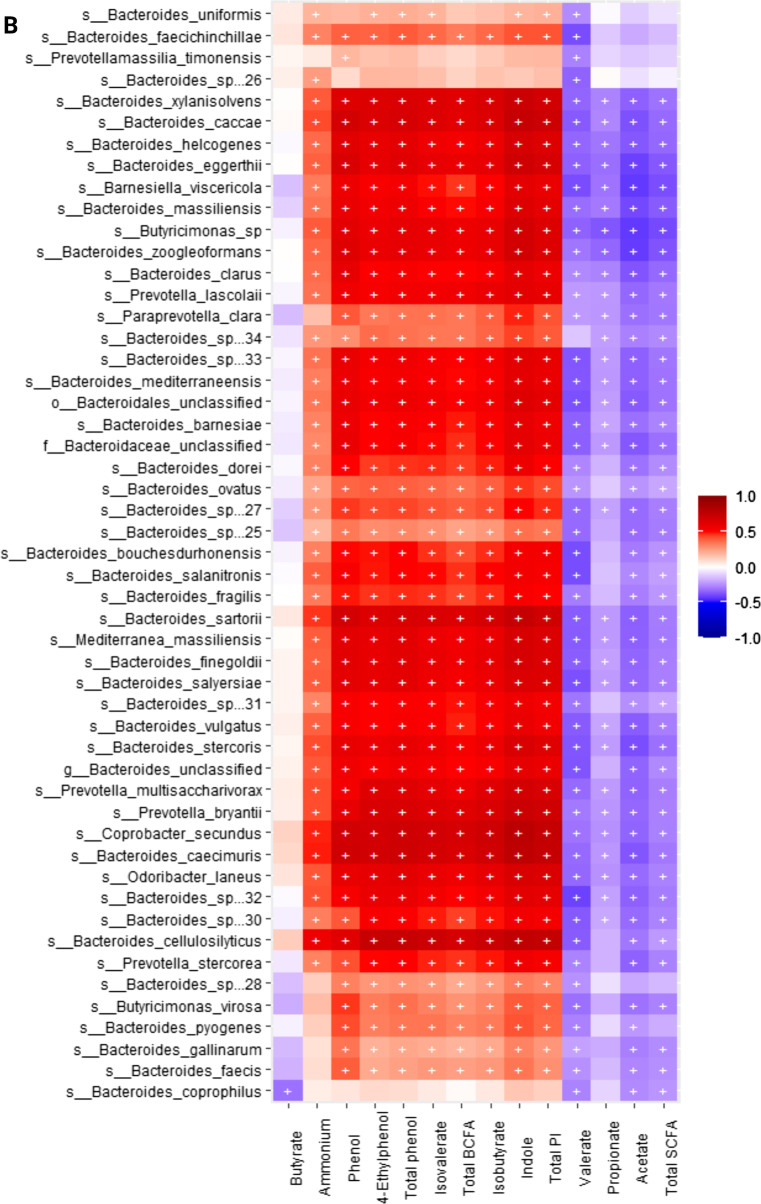

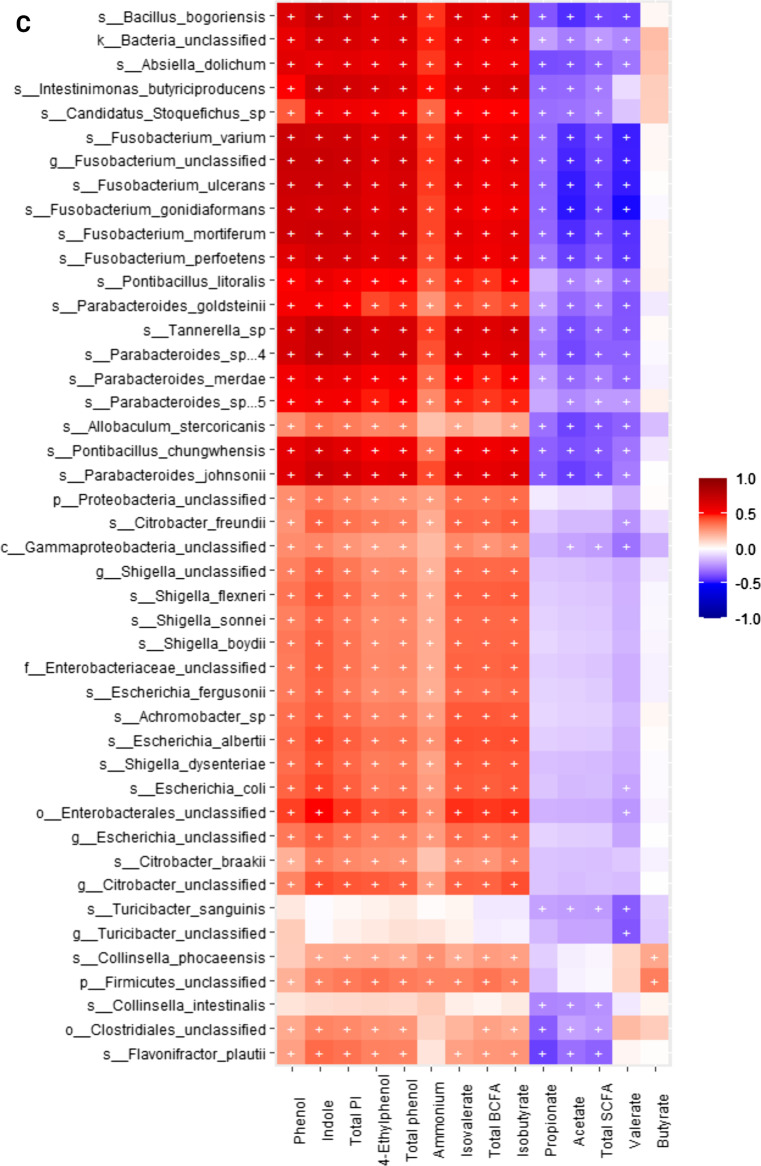

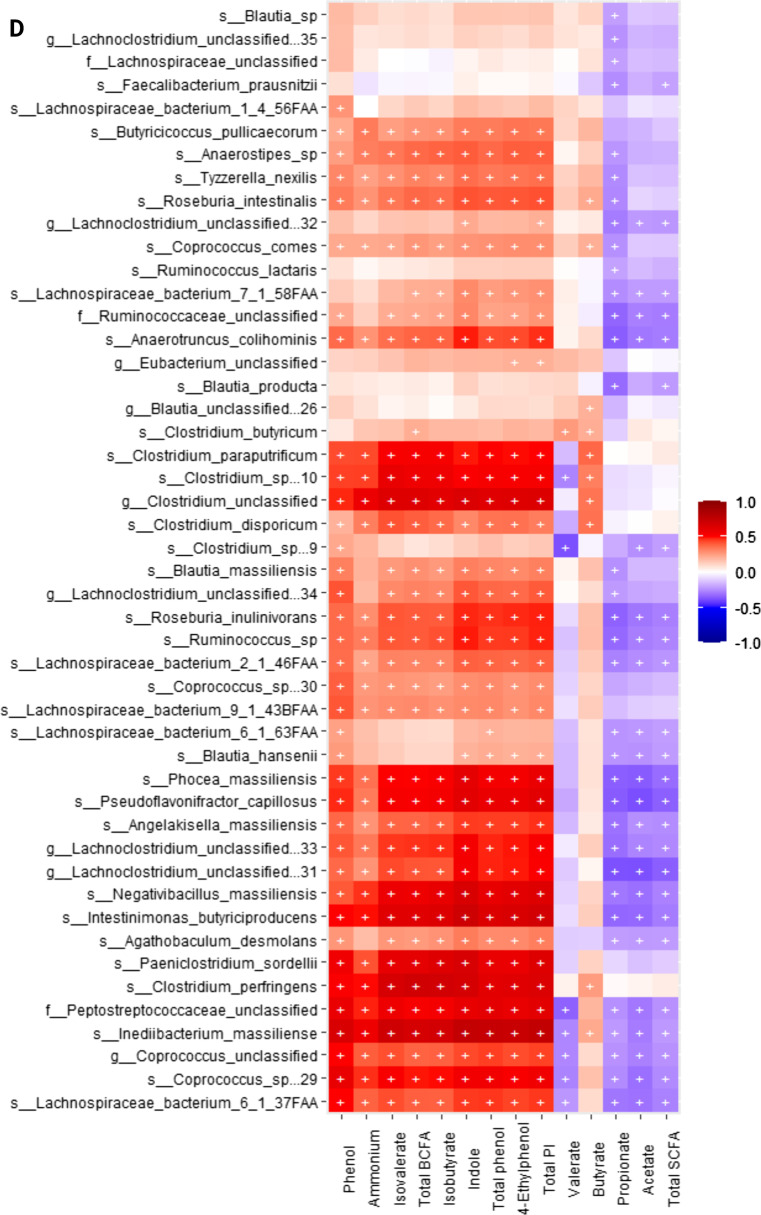
Heatmap of significant correlation values (r) between fecal microbial species and fecal metabolites. **A** Bacteria that primarily had a negative correlation with phenols, indoles and branched-chain fatty acids (BCFA; products of protein fermentation) and a positive correlation with short-chain fatty acids (SCFA; products of carbohydrate fermentation). **B-D** Bacteria that primarily had a positive correlation with phenols, indoles, and BCFA and a negative correlation with SCFA. The X and Y axes of the thermal graph are the metabolites and species, respectively. R values are represented by different colors (red: positive; blue: negative). Significant correlations (adj *P* < 0.05) are indicated by ‘+’

When conducting correlation analysis of fecal microbial taxa from shotgun data and fecal metabolite concentrations using Spearman’s rank correlation, we observed that the relative abundances of fecal *Blautia* spp., *Fournierella massiliensis*, *Lachnospiraceae bacterium*, *Holdemanella biformis*, *Romboutsia timonensis*, *Catenibacterium mitsuokai*, *Streptococcus* spp., *Prevotella bivia* and *P. copri*, and *Dorea* spp. were negatively (*P* < 0.05) correlated with fecal ammonium, isobutyrate, isovalerate, total BCFA, phenol, 4-ethylphenol, total phenol, indole, and total phenol and indole concentrations. The relative abundance of fecal *Megamonas* spp. were positively (*P* < 0.05) correlated with fecal acetate, propionate, and total SCFA, concentrations, and negatively (*P* < 0.05) correlated with fecal ammonium, isobutyrate, isovalerate, total BCFA, phenol, 4-ethylphenol, total phenol, indole, and total phenol and indole concentrations. *Lactobacillus* spp. and *Fusicatenibacter saccharivorans* were positively (*P* < 0.05) correlated with fecal acetate, propionate, valerate, and total SCFA concentrations, and negatively (*P* < 0.05) correlated with fecal fecal ammonium, isobutyrate, isovalerate, total BCFA, phenol, 4-ethylphenol, total phenol, indole, and total phenol and indole concentrations. *Clostridium* spp., *Blautia* spp., *Dorea* spp., *Lactobacillus* spp., *Coprococcus comes*, *Roseburia intestinalis*, *Collinsella phocaeensis*, *Ineddibacterium massiliense*, *Bifidobacterium pseudolongus*, *Catonella morbi*, and *Fusicatenibacter saccharivorans* were positively (*P* < 0.05) correlated with butyrate. (Fig. 9A**)**.

Additionally, the relative abundances of fecal *Bacteroides* spp., *Blautia* spp., *Fusobacterium* spp., *Lachnospiraceae bacterium*, *Parabacteroides* spp., *Pontibacillus* spp., *Prevotella* spp., *Roseburia* spp., *Tannerella* spp., *Butyricimonas* spp., *Clostridium* spp., *Coprococcus* spp., *Escherichia* spp., *Pontibacillus* spp., *Lachnospiraceae bacterium*, *Absiella dolichum*, *Bacillus bogoriensis*, *Barnesiella viscericola*, *Coprobacter secundus*, *Intestinimonas butyriciproducens*, *Odoribacter laneus*, *Mediterranea massiliensis*, *Barnesiella viscericola*, *Fusicatenibacter saccharivorans*, *Inediibacterium massiliense*, were positively (P < 0.05) correlated with fecal ammonium, isobutyrate, isovalerate, total BCFA, phenol, 4-ethylphenol, total phenol, indole, and total phenol and indole concentrations, and negatively (*P* < 0.05) correlated with fecal acetate, propionate, valerate, and total SCFA concentrations. Finally, *Shigella* spp. were positively (P < 0.05) correlated with fecal ammonium, isobutyrate, isovalerate, total BCFA, phenol, 4-ethylphenol, total phenol, indole, and total phenol and indole concentrations (Fig. 9B–D).

### Microbiome gene (KO term)-metabolome relationships

When conducting correlation analysis of fecal microbiome genes (KO terms) and fecal metabolite concentrations using Spearman’s rank correlation, we observed that the abundance of alcohol dehydrogenase (NADP+) was negatively (*P* < 0.05) correlated with ammonium. That gene belongs and contributes to the carbohydrate metabolism (*glycolysis / gluconeogenesis*, *pentose and glucuronate interconversions*, *ascorbate and aldarate metabolism*, and *pyruvate metabolism*), lipid metabolism (*glycerolipid metabolism*), and xenobiotic biodegradation and metabolism (*caprolactam degradation*) pathways (Additional file 1: Fig. S2).

## Discussion

Diet is known to have a strong influence on gastrointestinal health, fecal microbial populations, and fecal metabolite concentrations. Recent canine studies have investigated how dietary interventions affect gut microbial communities, but the longitudinal changes in gut microbial phylogeny, microbial function, and metabolite profiles immediately following a dietary change have been underexplored. To address these unknowns, the primary aim of this study was to determine when shifts in fecal microbial taxa, gene content, and metabolites occur and stabilize in dogs undergoing a drastic dietary change as well as describing what those changes are. A secondary aim was to identify correlations among fecal bacterial taxa and fecal metabolite concentrations.

To test our aims and identify whether longitudinal microbiome population shifts apply to dogs fed a variety of pet foods, diets greatly differing in ingredient composition, macronutrient content, and diet type (wet vs. dry) were chosen. An extruded diet rich in dietary fiber (i.e., HFD) was compared with a canned diet rich in fat and protein (i.e., CD). Dietary fiber and other non-digestible carbohydrates are the most studied macronutrients in this area because they are known to modulate the fecal microbiome composition and activity [3–8]. The amount and type of dietary proteins and fats may also affect the composition of fecal microbiota [12–16]. Another factor that has gained interest recently is the effect that processing and diet type have on the gut microbiota composition. Recent studies have examined the effects of wet (canned) or dry (kibbled) diet on fecal microbiota [31], with others comparing extruded, cooked, and/or raw diets on the fecal microbiota [32–34].

This study’s main focus was on the short-term responses of the fecal microbiota populations, microbial gene abundance, and fermentative metabolites to a rapid dietary change. In general, substantial changes occurred within a few days of diet change. In fact, random forest analysis was able to accurately differentiate the microbial compositions of dogs fed HFD or CD by d 16– only 2 d after diet change. The time by which stability was reached, however, took a few days longer and depended on the outcome (taxa, gene abundance, or metabolites shifted) and diet consumed. In general, fecal scores, dry matter percentage, and concentration of most fecal metabolites were shifted and stabilized by d 16. Fecal valerate concentration was the only exception. Fecal pH and alpha diversity measures appeared to be stable by d 20. The relative abundance of individual taxa and genes, however, varied from d 16 to d 24, depending on technology used (16S or shotgun) and outcome. The relative abundance of most microbial taxa were stable by d 16 to d 20 using shotgun sequencing, but was d 20 to d 24 for many of the taxa based on 16S data. The relative abundance of most microbial genes appeared to be stable by d 16 to d 20 for dogs fed the HFD, but took a little longer (d 20 to d 24) for those fed the CD.

Despite these minor differences, the data from this study demonstrate that fecal characteristics, metabolites, and microbial diversity, taxa, and gene content are all stable within 2 wk following a dietary change (d 27 of the current study). This result may have been expected because the microbiota present will immediately metabolize the nutrients received. This initial response will impact the metabolites and potentially stool characteristics soon after a dietary change. Over the long term, however, microbiota that have an advantage will become more prominent. This process will require more time to stabilize. With microbial taxa changes will come changes in gene content. These results are clinically relevant, as they indicate that a relatively short period of time is required for microbiota populations and activity to shift and stabilize following a dietary intervention, justifying the use of at least a 2–week time period prior to sample collection. Based on the data from the current study, that time frame is sufficient for identifying the effects of diet on the fecal microbiome and metabolites.

In humans, a short-term (3 d) macronutrient change (high-fat, high-protein diet vs. high-fiber intake) quickly affected the microbial populations and gene content associated with carbohydrate and protein fermentation. In that study, the relative abundance of bacterial genera such as *Alistipes*, *Bilophila*, and *Bacteroides* were decreased and the relative abundance of genera such as *Roseburia*, *Eubacterium rectale*, and *Ruminococcus bromii* were increased in individuals consuming the plant-based diet vs. animal-based diet [21]. Similar changes were noted in the current study, as the HFD quickly decreased *Bacteroides*, *Clostridium*, and *Fusobacterium* relative abundances and increased *Bifidobacterium*, *Blautia*, *Lactobacillus*, *Helicobacter*, and *Megamonas* relative abundances. Not surprisingly, microbial gene abundance was quickly and strongly affected by dietary change in the current study, with d 27 patterns for dogs fed both HFD and CD being clearly separated from d 13 (baseline prior to diet change). Because the diets tested were enriched in either fiber (HFD) or fat and protein (CD), it was not surprising to observe that microbial taxa and gene relative abundances shifted in opposite directions over time. The shifts observed were similar to that from humans eating plant-based vs. animal-based diets [21]. Fecal fermentative metabolites were rapidly impacted and stabilized by diet, with those coming from protein fermentation being increased rapidly by CD. Similar shifts have been noted in dogs high-protein, high-fat raw diets [34].

Most fecal microbiome and metabolite responses to HFD and CD diets were similar to what has been previously reported and were expected. For example, the relative abundance of Fusobacteriaceae was reduced and the relative abundance of Veillonellaceae was increased in animals fed HFD, and Clostridiaceae were enriched in animals fed CD. Feeding dietary fiber generally decreases Fusobacteria and increases Firmicutes in dogs [9]. Also, dogs fed high protein and/or meat typically have an increased relative abundance of fecal Clostridiaceae, *Slackia*, and Erysipelotrichaceae, whereas those fed a dry food with lower protein have increased the relative abundances of fecal *Faecalibacterium* and Veillonellaceae [33]. The relative abundances of *Bifidobacterium*, *Brachyspira*, *Clostridium*, *Helicobacter*, *Lactobacillus*, and *Megamonas*, and SCFA producers (*Dorea*; *Roseburia*; *Blautia*; *Prevotella*) were all enriched in animals fed HFD in the current study.

Many of the microbial shifts observed in dogs fed HFD were likely due to the greater availability and fermentation of non-digestible carbohydrates by the large intestinal microbiota. The SCFA, predominantly acetate, propionate, and butyrate, are known to be important to gastrointestinal health. Most of the SCFA produced in the large intestine are absorbed by the host and provide energy to intestinal epithelial cells [35–38]. An increase in lactate-producing bacteria such as *Bifidobacterium* and *Lactobacillus*, as that noted in dogs fed HFD, not only leads to increased production of lactate and other SCFA, but lowers intestinal pH and modulates gut immunity (reduced cytokine production), which are beneficial to the host. In the present study, *Lactobacillus* spp. were positively correlated with fecal acetate, propionate, valerate, and total SCFA concentrations.

Even though bifidobacteria and lactobacilli are often the focus of fiber- and prebiotic-containing diets, many other saccharolytic and SCFA-producing bacteria were increased in dogs fed the HFD, including *Blautia*, *Eubacterium*, *Megamonas*, *Lachnospira*, *Megasphaera*, *Prevotella*, and *Roseburia*, and were positively correlated with fecal SCFA concentrations. *Eubacterium* is associated with enhanced postprandial glucose and insulin response and improved health [39–44], while *Megamonas* is often associated with increased fecal butyrate, acetate, and propionate when greater dietary fibers are consumed [45–48]. In the present study, the relative abundance of fecal *Megamonas* spp. was positively correlated with fecal acetate, propionate, and total SCFA concentrations. Likewise, *Prevotella* is known to increase with greater non-digestible carbohydrate intake and partly responsible for the increase in fecal SCFA [49, 50]. In humans and mice, high fiber intake is associated with an increased relative abundance of gastrointestinal *Prevotella* [22, 51–53].

In the present study, *Clostridium* spp., *Blautia* spp., *Dorea* spp., *Lactobacillus* spp., *Coprococcus comes*, *Roseburia intestinalis*, *Collinsella phocaeensis*, *Ineddibacterium massiliense*, *Bifidobacterium pseudolongus*, *Catonella morbi*, and *Fusicatenibacter saccharivorans* relative abundances were positively correlated with fecal butyrate concentrations. Fecal butyrate has been reported previously to be positively correlated with *Blautia*, *Peptococcus*, and Coriobacteriaceae in dogs [54]. The positive correlation between fecal butyrate concentrations and members of the Coriobacteriaceae and Lachnospiraceae bacterial families suggest a positive role of these taxa on gastrointestinal health [54]. *Roseburia* spp. are also known to be a butyrate producers [55–57]. Not all bacterial groups increase in relative abundance or activity with high-fiber diets. In fact, the relative abundance of *Bacteroides* is often decreased in animals and humans consuming higher amounts of non-digestible fermentable carbohydrates, in part because they have a low tolerance for acidic conditions resulting from SCFA production [44, 58–62]. Therefore, the reduced relative abundance of *Bacteroides* in dogs fed HFD in the current study was expected.

Just as fecal SCFA concentrations increased in dogs fed HFD, protein catabolite concentrations (BCFA; phenols and indoles; ammonia) increased in the feces of dogs fed CD. This result was expected, as previous studies have reported such changes in dogs fed diets containing high protein concentrations or diets having low protein digestibility [33]. In the present study, the relative abundance of Clostridiaceae family members increased, while the relative abundance of Erysipelotrichaceae family members decreased in animals fed CD. In contrast, dogs fed HFD had reduced relative abundance of Fusobacteriaceae and Erysipelotrichaceae family members and increased relative abundance of Veillonellaceae family members. Many of these changes agree with those published previously, as dogs fed a diet high in red meat have been reported to have an increased relative abundances of fecal Clostridiaceae, *Dorea*, *Slackia*, Erysipelotrichaceae, and *Roseburia*, whereas dogs fed a commercial dry food with lower protein have increased relative abundances of fecal *Faecalibacterium* and Veillonellaceae [33]. Previous reports have also shown that higher dietary fiber intake generally decreases Fusobacteria and increases Firmicutes in dogs [9]. In regard to metabolites and microbiota, fecal acetate concentrations are often negatively correlated with *Escherichia*/*Shigella* and *Megamonas*, isovalerate is positively correlated with *Turicibacter*, and isobutyrate is positively correlated with *Blautia* and *Sutterella* [54]. In the present study, *Escherichia* were negatively correlated with fecal acetate, propionate, valerate, and total SCFA concentrations, *Blautia* were positively correlated with fecal ammonium, isobutyrate, isovalerate, total BCFA, phenol, 4-ethylphenol, total phenol, indole, and total phenol and indole concentrations. Relative abundance of *Megamonas* was positively correlated with fecal acetate, propionate, and total SCFA, concentrations, which partly agrees with a previous report showing that *Megamonas* was positively correlated with fecal propionate concentrations in humans [63].

In addition to the relationships between microbial taxa and metabolites, shotgun sequencing allowed the comparison of microbial gene (KO term) abundance and metabolite concentrations. In general, HFD increased the relative abundance of genes associated with carbohydrate, energy, and vitamin metabolism, and reduced genes associated with lipid metabolism. Carbohydrate and energy metabolism were positively affected by HFD, likely due to the higher intake of carbohydrates that serve as substrate and are associated with higher energy metabolism. Moreover, metabolites related to lipid metabolism were negatively affected by the consumption of HFD, which is aligned with the reduced fat intake (and likely fat reaching the colon) of those animals. Also, HFD had an increase in KO terms related to the metabolism of cofactors and vitamins (vitamin B6 metabolism). In the large intestine, *Bacteroides fragilis* and *Prevotella copri* (Bacteroidetes), *Bifidobacterium longum* and, *Collinsella aerofaciens* (Actinobacteria), and *Helicobacter pylori* (Proteobacteria) possess a vitamin B6 biosynthesis pathway [64]. In the present study, animals consuming HFD had an increase in *Bacteroides* spp., *Prevotella* spp., *Bifidobacterium* spp., and *Helicobacter* spp., in one or more time points compared with baseline, and this would be a feasible explanation for the increase of those metabolites. Overall, CD increased the relative abundance of genes associated with energy and amino acid metabolism, and reduced the relative abundance of genes associated with starch and sucrose metabolism. The reduction in genes associated with starch and sucrose metabolism is aligned with the lower carbohydrate intake of the animals consuming that diet. Amino acid and energy metabolism were positively affected by CD, especially sulfur and nitrogen metabolism, which is in line with the higher intake of protein and fat of those animals.

Next-generation sequencing methods have been widely used to characterize gut microbiota populations in recent years [65], with the most popular strategies including 16S rRNA gene amplicon-based sequencing or shotgun sequencing [66–68]. Although most of the canine microbiota data published to date has come from 16S rRNA gene-based sequencing, its major limitation is that taxa are assigned based on the sequence of a single region of the bacterial genome, with primer selection being very important because some are known to over- or under-represent specific taxa [69]. Shotgun sequencing allows for a more accurate definition at the species level and allows the identification of a larger number of taxa [70]. In the present study, both 16S rRNA gene and shotgun sequencing were used to identify how different the results were between methods. While some taxonomic differences were noted, the overall shifts were generally in agreement between shotgun and 16S formats.

## Conclusions

In this study, we demonstrate that an abrupt dietary change results in a rapid change to and stabilization of fecal characteristics, metabolites, and microbial diversity, taxa, and gene content of dogs. In general, substantial changes occurred within a few days of diet change, with random forest analysis accurately differentiating the microbiota of dogs fed different diets after only 2 days. The speed by which stability was reached took a few days longer and depended on the outcome (taxa, gene abundance, or metabolites shifted) and diet consumed, but usually within 6–10 days. Despite the minor differences that occurred among the outcomes, our data demonstrate that fecal characteristics, metabolites, and microbial diversity, taxa, and gene content are all stable within 2 wk following a dietary change. As expected, dogs changed to a high-fiber diet quickly had a lower fecal pH, higher fecal SCFA concentrations, higher relative abundance of bacterial genera known to break down fiber and/or produce SCFA, and higher abundance of genes associated with carbohydrate breakdown. In contrast, dogs changed to a high-protein, high-fat canned diet had higher protein catabolite (BCFA; phenols and indoles; ammonia) concentrations and proteolytic bacteria. Further advancements are needed using shotgun sequencing so that species-specific resolution may be achieved. Moreover, future research must expand into client-owned animals that live in a variety of settings so that environmental influences may be accounted for.

## Materials and methods

### Animals, diets, and treatments

Twelve adult female beagles (mean age: 5.16 ± 0.87 year; mean BW: 13.37 ± 0.68 kg) were used and housed individually in pens (1.0 m wide × 1.8 m long) in a humidity- and temperature-controlled room on a 14 h light: 10 h dark cycle. Dogs had access to fresh water *ad libitum* at all times and were fed once a day in the morning to maintain BW throughout the study. Dogs were weighed and body condition scores were assessed using a 9-point scale [71] weekly before feeding. All animal care and experimental procedures were approved by the University of Illinois Institutional Animal Care and Use committee (Protocol No. 17,276) prior to experimentation.

A crossover design was conducted. Each 28-d experimental period consisted of an adaptation phase (d 1–14) and a diet transition phase (d 15–28). Diets that met all Association of American Feed Control Officials (AAFCO, 2017) nutrient recommendations for adult dogs at maintenance were fed, including (1) a baseline control diet (a dry kibble experimental diet); (2) a commercial CD (Ol’ Roy Cuts in Gravy with Savory Beef; Walmart, Bentonville, AR; Table 1); and (3) a HFD that was composed of the experimental diet plus 22.5 g/d of soluble corn fiber (Nutriose® Soluble Digestion-Resistant Prebiotic Corn Fiber; Roquette America Inc., Geneva, IL) that was top-dressed on the diet just prior to feeding.

**Table 1:** Analyzed chemical composition of the experimental diet and canned diet fed to dogs

Item	Experimental Diet		Canned Diet^1^	
Dry matter (DM; %)	92.82		23.90	
	%, of DM	g/1,000 kcal	% DM	g/1,000 kcal
Crude protein	28.30	80.6	41.27	105.6
Acid-hydrolyzed fat	14.36	40.9	24.35	62.3
Total dietary fiber	15.98	45.5	11.72	30.0
Ash	5.95	---	12.96	---
Nitrogen-free extract^a^	35.41	100.9	9.72	24.9
Metabolizable energy^b^ (kcal/g)	3.51	---	3.91	---

^a^Nitrogen-free extract (%) = 100% - (crude protein % + acid-hydrolyzed fat % + total dietary fiber % + ash %)

^b^Metabolizable energy = 3.5 kcal/g ⋅ crude protein (%) + 8.5 kcal/g ⋅ acid-hydrolyzed fat (%) + 3.5 kcal/g ⋅ nitrogen-free extract (%)

^1^Ingredient: water sufficient for processing, chicken, meat by-products, wheat flour, liver, beef, wheat gluten, chicken meal, potato starch, cornstarch, guar gum, added color, salt, sodium phosphate, potassium chloride, vegetable oil, vitamins (vitamin E supplement, thiamine mononitrate, niacin supplement, D-calcium pantothenate, vitamin a supplement, riboflavin supplement, biotin, vitamin B12 supplement, pyridoxine hydrochloride, vitamin D3 supplement, folic acid), choline chloride, minerals (ferrous sulfate, zinc oxide, copper proteinate, sodium selenite, manganese sulfate, potassium iodide), onion powder, garlic powder

### Fecal sample collection

On d 13, 16, 20, 24, and 27 of each experimental period, fresh fecal samples from each dog were collected within 15 min of defecation. Fecal samples were scored according to a 5-point scale: 1 = hard, dry pellets, small hard mass; 2 = hard, formed, dry stool; remains firm and soft; 3 = soft, formed, and moist stool, retains shape; 4 = soft, unformed stool, assumes shape of container; and 5 = watery, liquid that can be poured. Fecal pH was measured immediately using an AP10 pH meter (Denver Instrument, Bohemia, NY) equipped with a Beckman Electrode (Beckman Instruments Inc., Fullerton, CA) prior to collecting aliquots for DM, metabolite, and microbiome measurements.

An aliquot of fresh feces was dried at 105 °C for 2 d for DM determination. Aliquots for analysis of phenols and indoles were frozen at -20 °C immediately after collection. One aliquot was collected and placed in approximately 2 mL of 2 N hydrochloric acid for ammonia, SCFA, and BCFA analyses. Fresh fecal samples for microbiome analysis were collected into 2.0 mL cryogenic vials, immediately snap-frozen in liquid nitrogen, and stored at − 80 °C until analysis.

### Fecal metabolites

Fecal SCFA and BCFA concentrations were determined by gas chromatography according to Erwin et al. (1961) using a gas chromatograph (Hewlett-Packard 5890 A series II, Palo Alto, CA) and a glass column (180 cm × 4 mm i.d.) packed with 10% SP-1200/1% H_3_PO_4_ on 80/100 + mesh Chromosorb WAW (Supelco Inc., Bellefonte, PA). Nitrogen was the carrier with a flow rate of 75 mL/min. Oven, detector, and injector temperatures will be 125, 175, and 180 °C, respectively. Fecal ammonia concentrations were determined according to the method of Chaney and Marbach (1962). Fecal phenol and indole concentrations were determined using gas chromatography according to the methods described by Flickinger et al. (2003).

### Fecal DNA extraction, 16S rRNA gene sequencing, and data analyses

Total DNA from fecal samples were extracted using Mo-Bio PowerSoil kits (MO BIO Laboratories, Inc., Carlsbad, CA). The concentration of extracted DNA was quantified using a Qubit 3.0 Fluorometer (Life Technologies, Grand Island, NY). 16S rRNA gene amplicons were generated using a Fluidigm Access Array (Fluidigm Corporation, South San Francisco, CA) in combination with Roche High Fidelity Fast Start Kit (Roche, Indianapolis, IN). The primers 515 F (5′-GTGCCAGCMGCCGCGGTAA-3′) and 806R (5′-GGACTACHVGGGTWTCTAAT-3′) that target a 252 bp-fragment of the V4 region (291 bp including primers) of the 16S rRNA gene were used for amplification (primers synthesized by IDT Corp., Coralville, IA) [76]. CS1 forward tag and CS2 reverse tag were added according to the Fluidigm protocol. The quality of the amplicons were assessed using a Fragment Analyzer (Advanced Analytics, Ames, IA) to confirm amplicon regions and sizes. A DNA pool was generated by combining equimolar amounts of the amplicons from each sample. The pooled samples were then size selected on a 2% agarose E-gel (Life technologies, Grand Island, NY) and extracted using a Qiagen gel purification kit (Qiagen, Valencia, CA). Cleaned size-selected pooled products were run on an Agilent Bioanalyzer to confirm appropriate profile and average size. Illumina sequencing was performed on a MiSeq using v3 reagents (Illumina Inc., San Diego, CA) at the W. M. Keck Center for Biotechnology at the University of Illinois.

16S rRNA gene sequencing data were processed using QIIME 2 (version 2018.8; Bolyen et al., 2019). Trimmomatic (version 0.36) was used to remove sequencing adaptors (Bolger et al., 2014) and then imported into the QIIME2 environment. DADA2 was used to remove low-quality reads, denoise, and filter chimeras. Amplicon sequence variants were generated using DADA2 and then taxonomically assigned using scikit-learn classifier (Callahan et al., 2016) against the Greengenes 13_8 reference database (Desantis et al., 2006). An even sampling depth of 24,423 sequences was used for assessing alpha- and beta-diversity measures. Alpha diversity was calculated using phylogenetic diversity (PD) whole tree, Pielou’s evenness, and Shannon diversity index. Beta diversity was calculated using weighted and unweighted UniFrac (Lozupone and Knight, 2005) distance measures and presented by principal coordinates analysis (PCoA) plots.

### Metagenomics sequencing and data analyses

Libraries were prepared with a procedure adapted from the Nextera DNA Library Prep (Illumina, USA). Shotgun metagenomic sequencing was performed with BoosterShot™ (Shallow Sequencing, 2 M reads/sample) at Diversigen Inc., USA as previously described [77]. Libraries were sequenced on an Illumina NovaSeq 6000 using single-end 1 × 100 reads (Illumina, USA). Library controls included a no template control (water) and DNA from a characterized homogenized stool.

For quality control, single end shotgun reads were trimmed and processed using Shi7 [78]. The sequences were then aligned to the NCBI RefSeq representative prokaryotic genome collection at 97% identity with BURST using default settings [79]. A total of 70,326,694 reads were mappable and the mean sequencing depth per sample was 1.17 million. Taxa present in < 5% of the samples were removed. The resulting taxonomy table was also aggregated at higher taxonomic levels (e.g., species, genus, family).

Kyoto Encyclopedia of Genes and Genomes Orthology groups (KEGG KO) were observed directly using alignment at 97% identity against a gene database derived from the genomic database used above. The KO table contained the directly observed KO counts within each sample. KO terms present in < 5% of the samples were removed as part of the quality filtering process.

Species richness and Shannon’s diversity indices were computed by rarefying samples to various depths starting from 25,000 to 950,000 sequences per sample and increasing sequence depth by 25,000 reads. One hundred iterations were performed at each depth and the mean values were used as the estimate of these measures in each sample. To investigate the effect of dietary treatments on alpha-diversity, the species richness and Shannon Index were calculated using the rarefaction depth of 950,000. The Wilcoxon signed rank test was used to compare changes of alpha diversity metrics among treatments.

The non-rarefied count data were log-transformed and principal coordinate analysis (PCoA) was performed in R using the Bray-Curtis and Jensen-Shannon distances calculated with the vegan package at the species level [80]. Permutational multivariate analysis of variance (PERMANOVA) was performed using Bray-Curtis distance with 10,000 randomizations to assess the differences in community composition using the vegan package [80]. Differential abundance of bacterial phyla, species, and KO terms between treatments was assessed using a negative binomial generalized linear model using the differential expression analysis for sequence count data version 2 (DESeq2) package (Love et al., 2014). Taxa with absolute log2 (fold change [FC]) > 2 and adjusted *P* < 0.05 were considered significant. The Benjamini Hochberg method was performed to control the false discovery rate due to multiple comparisons.

### Clustering

Partitioning around medoids clustering was performed using the *cluster* package [81]. Individuals were clustered into multiple clusters (K = 1 to 5) based on the top two PCoA dimensions obtained using Bray-Curtis distances. Goodness of clustering was assessed using a “gap” statistic with 1000 bootstrapped replicates.

### Random forest models

Random forest classifiers [82] were constructed using the repeated k-fold cross validation and random search implemented in R-package *caret* [83]. The data were partitioned into training and validation sets containing 70% and 30% of the samples respectively and species were used as the predictors. The models were trained by optimizing the tuning parameters using 10-fold cross validation repeated 3 times and accuracy was used to select the optimal model. The performance of the classifiers was assessed by generating area under the receiver operating characteristic curves using the R-package *ROCR* [84]. Variable importances were calculated using the default *varImp* function in the *caret* package.

### Statistical analyses

All physiological data were analyzed using the Mixed Models procedure of SAS (version 9.4; SAS Institute, Inc., Cary, NC), with treatment considered as a fixed effect and dog and period considered random effects. Data were tested for normality using the UNIVARIATE procedure of SAS. Differences between treatments were determined using a Fisher-protected least significant difference with a Tukey adjustment to control for experiment-wise error. A probability of *P* ≤ 0.05 was accepted as statistically significant and 0.05 < *P* ≤ 0.10 was set as trends. Reported pooled standard errors of the mean (SEM) were determined according to the Mixed Models procedure of SAS. The similarity of the microbiota/KO terms and metabolites profiles were assessed by Spearman’s rank correlation coefficient (r) carried out using R (R version 4.0.5), with a p.adj. threshold of 0.05.

## Supplementary Information

**Additional file 1: Table S1.** Bacterial species (from shotgun data) altered in dogs fed the high-fiber diet on days (D) 16, 20, 24, and 27. **Table S2.** Bacterial species (from shotgun data) altered in dogs fed the high-fiber diet on days (D) 16 and 20; 16, 20, and 24; and 24 and 27. **Table S3.** Bacterial species (from shotgun data) altered in dogs fed the high-fiber diet on days (D) 20, 24, and 27. **Table S4.** Bacterial species (from shotgun data) altered in dogs fed the protein-rich canned diet on days (D) 16, 20, 24, and 27. **Table S5.** Bacterial species (from shotgun data) altered in dogs fed the protein-rich canned diet on days (D) 16, 20, and 24; and 24 and 27. **Table S6.** Bacterial species (from shotgun data) altered in dogs fed the protein-rich canned diet on days (D) 20, 24, and 27. **Table S7.** Bacterial phyla and genera (% of total sequences) from 16S rRNA sequencing in feces of dogs fed a high-fiber or protein-rich canned diet on days (D) 13, 16, 20, 24, and 27. **Table S8.** Bacterial gene (KO term) abundance that increased in feces of dogs fed the high-fiber diet. **Table S9.** Bacterial gene (KO term) abundance that decreased in feces of dogs fed the high-fiber diet. **Table S10.** Bacterial gene (KO term) abundance that were altered in feces of dogs fed the protein-rich canned diet. **Figure S1.** Fecal microbiota communities of dogs fed a high-fiber diet or protein-rich canned diet from 16S rRNA sequencing. Alpha diversity measures: phylogenetic diversity (A), Shannon diversity index (B), and Pielou’s evenness (C). Beta diversity measures show distinct separation of dogs transitioned to HFD and CD were noted by d 20 for unweighted Unifrac distance (D) and d 16 for weighted Unifrac distance (E). *Mean values within time points were different between diets (P < 0.05); #Mean values within time points tended to be different between diets (P < 0.10). **Figure S2.** Heatmap of significant correlation values (r) between fecal KO terms and fecal metabolites. The X and Y axes of the thermal graph are the metabolites and KO terms, respectively. R values are represented by different colors (red: positive; blue: negative). Significant correlations (adj P < 0.05) are indicated by ‘+’.

## Data Availability

The whole-genome shotgun sequence reads used for this study are available as NCBI Short Read Archive objects associated with the NCBI BioProject PRJNA846742.

## Abbreviations

BCFA: Branched-chain fatty acids.
BW: Body weight.
CD: Canned diet.
DESeq2: Differential expression analysis for sequence count data version 2.
DM: Dry matter.
HFD: High-fiber diet.
KO: KEGG orthology.
PCoA: Principal coordinates analysis.
PD: Phylogenetic diversity.
PERMANOMA: Permutational multivariate analysis of variance.
SCFA: Short-chain fatty acids.
