# Supplementary material for: Longitudinal fecal microbiome and metabolite data demonstrate rapid shifts and subsequent stabilization after an abrupt dietary change in healthy adult dogs
Source: Anim Microbiome. 2022 Aug 1;4:46. doi: 10.1186/s42523-022-00194-9 (PMC9341101; doi:10.1186/s42523-022-00194-9)
Supplement: Supplementary file 1 — Additional file 1: Table S1. Bacterial species (from shotgun data) altered in dogs fed the high-fiber diet on days (D) 16, 20, 24, and 27. Table S2. Bacterial species (from shotgun data) altered in dogs fed the high-fiber diet on days (D) 16 and 20; 16, 20, and 24; and 24 and 27. Table S3. Bacterial species (from shotgun data) altered in dogs fed the high-fiber diet on days (D) 20, 24, and 27. Table S4. Bacterial species (from shotgun data) altered in dogs fed the protein-rich canned diet on days (D) 16, 20, 24, and 27. Table S5. Bacterial species (from shotgun data) altered in dogs fed the protein-rich canned diet on days (D) 16, 20, and 24; and 24 and 27. Table S6. Bacterial species (from shotgun data) altered in dogs fed the protein-rich canned diet on days (D) 20, 24, and 27. Table S7. Bacterial phyla and genera (% of total sequences) from 16S rRNA sequencing in feces of dogs fed a high-fiber or protein-rich canned diet on days (D) 13, 16, 20, 24, and 27. Table S8. Bacterial gene (KO term) abundance that increased in feces of dogs fed the high-fiber diet. Table S9. Bacterial gene (KO term) abundance that decreased in feces of dogs fed the high-fiber diet. Table S10. Bacterial gene (KO term) abundance that were altered in feces of dogs fed the protein-rich canned diet. Figure S1. Fecal microbiota communities of dogs fed a high-fiber diet or protein-rich canned diet from 16S rRNA sequencing. Alpha diversity measures: phylogenetic diversity (A), Shannon diversity index (B), and Pielou’s evenness (C). Beta diversity measures show distinct separation of dogs transitioned to HFD and CD were noted by d 20 for unweighted Unifrac distance (D) and d 16 for weighted Unifrac distance (E). *Mean values within time points were different between diets (P < 0.05); #Mean values within time points tended to be different between diets (P < 0.10). Figure S2. Heatmap of significant correlation values (r) between fecal KO terms and fecal metabolites. The X and Y axes of the thermal g [file 42523_2022_194_MOESM1_ESM.docx]

**Supplementary Table 1.** Bacterial species (from shotgun data) altered in dogs fed the high-fiber diet on days (D) 16, 20, 24, and 27

| Days | log2 | Phylum | Family | Genus | Species |
| --- | --- | --- | --- | --- | --- |
| D13_20 | 3.19 | Firmicutes | Lactobacillaceae | *Lactobacillus* | *Lactobacillus helveticus* |
| D13_16 | 3.65 |  |  |  |  |
| D13_24 | 4.06 |  |  |  |  |
| D13_27 | 5.23 |  |  |  |  |
| D13_20 | 2.71 | Firmicutes | Lactobacillaceae | *Lactobacillus* | *Lactobacillus parabuchneri* |
| D13_16 | 2.77 |  |  |  |  |
| D13_27 | 4.22 |  |  |  |  |
| D13_24 | 5.15 |  |  |  |  |
| D13_20 | -4.54 | Firmicutes | Clostridiaceae | *Clostridium* | *Clostridium polynesiense* |
| D13_27 | -3.91 |  |  |  |  |
| D13_24 | -3.42 |  |  |  |  |
| D13_16 | -2.88 |  |  |  |  |
| D13_20 | -3.03 | Firmicutes | Lachnospiraceae |  | Lachnospiraceae bacterium |
| D13_24 | -2.5 |  |  |  |  |
| D13_27 | -2.35 |  |  |  |  |
| D13_16 | -2.11 |  |  |  |  |
| D13_16 | 3.23 | Firmicutes | Lachnospiraceae | *Blautia* | *[Ruminococcus] gnavus* |
| D13_27 | 3.8 |  |  |  |  |
| D13_20 | 3.96 |  |  |  |  |
| D13_24 | 4.11 |  |  |  |  |
| D13_16 | 2.49 | Firmicutes | Lachnospiraceae | *Fusicatenibacter* | *Fusicatenibacter saccharivorans* |
| D13_24 | 2.88 |  |  |  |  |
| D13_27 | 3.21 |  |  |  |  |
| D13_20 | 3.77 |  |  |  |  |
| D13_16 | 2.43 | Firmicutes | Ruminococcaceae | *Fournierella* | *Fournierella massiliensis* |
| D13_20 | 3.34 |  |  |  |  |
| D13_27 | 3.37 |  |  |  |  |
| D13_24 | 3.72 |  |  |  |  |
| D13_20 | -3.46 | Firmicutes | Ruminococcaceae | *Negativibacillus* | *Negativibacillus massiliensis* |
| D13_24 | -2.96 |  |  |  |  |
| D13_27 | -2.5 |  |  |  |  |
| D13_16 | -2.33 |  |  |  |  |
| D13_20 | -2.51 | Firmicutes | Ruminococcaceae | *Phocea* | *Phocea massiliensis* |
| D13_24 | -2.32 |  |  |  |  |
| D13_27 | -2.18 |  |  |  |  |
| D13_16 | -2.08 |  |  |  |  |
| D13_27 | -2.71 | Firmicutes | Erysipelotrichaceae | *Absiella* | *Absiella dolichum* |
| D13_20 | -2.67 |  |  |  |  |
| D13_24 | -2.35 |  |  |  |  |
| D13_16 | -2.25 |  |  |  |  |
| D13_16 | 3.19 | Firmicutes | Erysipelotrichaceae | *Catenibacterium* | *Catenibacterium mitsuokai* |
| D13_20 | 4.14 |  |  |  |  |
| D13_24 | 4.76 |  |  |  |  |
| D13_27 | 4.85 |  |  |  |  |
| D13_16 | 2.1 | Firmicutes | Erysipelotrichaceae | *Erysipelatoclostridium* | *[Clostridium] saccharogumia* |
| D13_20 | 2.4 |  |  |  |  |
| D13_24 | 2.41 |  |  |  |  |
| D13_27 | 2.54 |  |  |  |  |
| D13_27 | 2 | Firmicutes | Erysipelotrichaceae | *Erysipelatoclostridium* | *[Clostridium] spiroforme* |
| D13_24 | 2.06 |  |  |  |  |
| D13_16 | 2.13 |  |  |  |  |
| D13_20 | 2.3 |  |  |  |  |
| D13_16 | 2.47 | Firmicutes | Erysipelotrichaceae | *Kandleria* | *Kandleria vitulina* |
| D13_20 | 3.55 |  |  |  |  |
| D13_24 | 4.15 |  |  |  |  |
| D13_27 | 4.26 |  |  |  |  |
| D13_24 | -3.67 | Firmicutes | Acidaminococcaceae | *Acidaminococcus* | *Acidaminococcus intestini* |
| D13_20 | -2.93 |  |  |  |  |
| D13_27 | -2.85 |  |  |  |  |
| D13_16 | -2.21 |  |  |  |  |
| D13_24 | 3.1 | Firmicutes | Selenomonadaceae | *Megamonas* |  |
| D13_20 | 3.1 |  |  |  |  |
| D13_16 | 3.12 |  |  |  |  |
| D13_27 | 3.29 |  |  |  |  |
| D13_24 | 2.34 | Firmicutes | Selenomonadaceae | *Megamonas* | *Megamonas funiformis* |
| D13_20 | 2.38 |  |  |  |  |
| D13_16 | 2.47 |  |  |  |  |
| D13_27 | 2.57 |  |  |  |  |
| D13_24 | 2.09 | Firmicutes | Selenomonadaceae | *Megamonas* | *Megamonas rupellensis* |
| D13_20 | 2.24 |  |  |  |  |
| D13_27 | 2.29 |  |  |  |  |
| D13_16 | 2.4 |  |  |  |  |
| D13_24 | 3.17 | Firmicutes | Selenomonadaceae | *Megamonas* | *Megamonas sp.* |
| D13_16 | 3.18 |  |  |  |  |
| D13_20 | 3.19 |  |  |  |  |
| D13_27 | 3.35 |  |  |  |  |
| D13_24 | 2.37 | Proteobacteria | Helicobacteraceae | *Helicobacter* |  |
| D13_16 | 2.63 |  |  |  |  |
| D13_20 | 2.63 |  |  |  |  |
| D13_27 | 4.36 |  |  |  |  |
| D13_24 | 2.18 | Proteobacteria | Helicobacteraceae | *Helicobacter* | *Helicobacter bilis* |
| D13_16 | 2.59 |  |  |  |  |
| D13_20 | 2.63 |  |  |  |  |
| D13_27 | 4.31 |  |  |  |  |

^*^log2 = log 2 fold change compared to baseline (D13).

**Supplementary Table 2.** Bacterial species (from shotgun data) altered in dogs fed the high-fiber diet on days (D) 16 and 20; 16, 20, and 24; and 24 and 27

| Days | log2* | Phylum | Family | Genus | Species |
| --- | --- | --- | --- | --- | --- |
| D13_20 | 2.79 | Bacteroidetes | Rikenellaceae | *Alistipes* | *Alistipes sp.* |
| D13_16 | 2.87 |  |  |  |  |
| D13_16 | -2.62 | Proteobacteria | Campylobacteraceae | *Campylobacter* | *Campylobacter fetus* |
| D13_20 | -2.56 |  |  |  |  |
| D13_20 | -2.93 | Firmicutes |  | *Intestinimonas* | *Intestinimonas butyriciproducens* |
| D13_24 | -2.38 |  |  |  |  |
| D13_16 | -2.11 |  |  |  |  |
| D13_24 | 2.34 | Actinobacteria | Streptomycetaceae | *Streptomyces* | *Streptomyces sampsonii* |
| D13_27 | 2.74 |  |  |  |  |
| D13_27 | -2.64 | Bacteroidetes | Bacteroidaceae | *Bacteroides* | *Bacteroides paurosaccharolyticus* |
| D13_24 | -2.23 |  |  |  |  |
| D13_24 | 2.87 | Bacteroidetes | Bacteroidaceae | *Bacteroides* | *Bacteroides sp.* |
| D13_27 | 3.47 |  |  |  |  |
| D13_24 | 2.04 | Bacteroidetes | Prevotellaceae | *Prevotella* | *Prevotella bergensis* |
| D13_27 | 2.94 |  |  |  |  |
| D13_27 | 3.33 | Cyanobacteria | Nostocaceae | *Nostoc* | *Nostoc linckia* |
| D13_24 | 3.42 |  |  |  |  |
| D13_27 | -2.34 | Firmicutes | Bacillaceae | *Bacillus* | *Bacillus bogoriensis* |
| D13_24 | -2.17 |  |  |  |  |
| D13_27 | -3.36 | Firmicutes | Clostridiaceae | *Clostridium* | *Clostridium baratii* |
| D13_24 | -2.74 |  |  |  |  |
| D13_24 | -4.07 | Firmicutes | Clostridiaceae | *Clostridium* | *Clostridium disporicum* |
| D13_27 | -3.65 |  |  |  |  |
| D13_27 | 2.45 | Firmicutes | Clostridiaceae | *Clostridium* | *Clostridium sp.* |
| D13_24 | 2.48 |  |  |  |  |
| D13_27 | 2.76 | Firmicutes | Clostridiaceae | *Clostridium* | *Clostridium sp.* |
| D13_24 | 2.81 |  |  |  |  |
| D13_27 | 2.55 | Firmicutes | Lachnospiraceae |  | Lachnospiraceae bacterium |
| D13_24 | 2.9 |  |  |  |  |
| D13_27 | 2.31 | Firmicutes | Lachnospiraceae | *Anaerostipes* | *Anaerostipes hadrus* |
| D13_24 | 2.33 |  |  |  |  |
| D13_24 | -2.76 | Firmicutes | Lachnospiraceae | *Butyrivibrio* |  |
| D13_27 | -2.13 |  |  |  |  |
| D13_24 | 2.75 | Firmicutes | Lachnospiraceae | *Lachnoclostridium* | *[Clostridium] aminophilum* |
| D13_27 | 3.65 |  |  |  |  |
| D13_24 | 2.55 | Firmicutes | Lachnospiraceae | *Lachnospira* | *Lachnospira multipara* |
| D13_27 | 2.77 |  |  |  |  |
| D13_24 | 2.28 | Firmicutes | Erysipelotrichaceae |  | Erysipelotrichaceae bacterium |
| D13_27 | 3.06 |  |  |  |  |
| D13_27 | 2.12 | Firmicutes | Erysipelotrichaceae | *Faecalitalea* | *Faecalitalea cylindroides* |
| D13_24 | 2.61 |  |  |  |  |
| D13_24 | 2.23 | Firmicutes | Erysipelotrichaceae | *Massiliomicrobiota* | *Massiliomicrobiota timonensis* |
| D13_27 | 2.23 |  |  |  |  |
| D13_24 | -2.36 | Firmicutes | Erysipelotrichaceae | *Turicibacter* |  |
| D13_27 | -2.02 |  |  |  |  |
| D13_27 | -2.79 | Fusobacteria | Fusobacteriaceae | *Fusobacterium* | *Fusobacterium gonidiaformans* |
| D13_24 | -2.45 |  |  |  |  |
| D13_27 | -2.48 | Fusobacteria | Fusobacteriaceae | *Fusobacterium* | *Fusobacterium nucleatum* |
| D13_24 | -2.21 |  |  |  |  |
| D13_27 | -2.35 | Fusobacteria | Fusobacteriaceae | *Fusobacterium* | *Fusobacterium periodonticum* |
| D13_24 | -2.04 |  |  |  |  |
| D13_24 | 3.43 | Proteobacteria |  |  |  |
| D13_27 | 3.85 |  |  |  |  |

^*^log2 = log 2 fold change compared to baseline (D13).

**Supplementary Table 3.** Bacterial species (from shotgun data) altered in dogs fed the high-fiber diet on days (D) 20, 24, and 27

| Days | log2* | Phylum | Family | Genus | Species |
| --- | --- | --- | --- | --- | --- |
| D13_27 | 5.69 | Actinobacteria | Bifidobacteriaceae | *Bifidobacterium* | *Bifidobacterium boum* |
| D13_24 | 6.09 |  |  |  |  |
| D13_20 | 6.31 |  |  |  |  |
| D13_24 | 2.73 | Actinobacteria | Bifidobacteriaceae | *Bifidobacterium* | *Bifidobacterium pseudolongum* |
| D13_27 | 3.12 |  |  |  |  |
| D13_20 | 3.88 |  |  |  |  |
| D13_27 | 5.87 | Actinobacteria | Bifidobacteriaceae | *Bifidobacterium* | *Bifidobacterium thermophilum* |
| D13_24 | 6.02 |  |  |  |  |
| D13_20 | 6.37 |  |  |  |  |
| D13_27 | -2.64 | Bacteroidetes | Bacteroidaceae | *Bacteroides* | *Bacteroides cellulosilyticus* |
| D13_24 | -2.21 |  |  |  |  |
| D13_20 | -2.05 |  |  |  |  |
| D13_27 | -3.69 | Bacteroidetes | Bacteroidaceae | *Bacteroides* | *Bacteroides sp.* |
| D13_20 | -3.08 |  |  |  |  |
| D13_24 | -2.71 |  |  |  |  |
| D13_24 | -3.55 | Bacteroidetes | Porphyromonadaceae | *Petrimonas* | *Petrimonas mucosa* |
| D13_27 | -3.52 |  |  |  |  |
| D13_20 | -2.81 |  |  |  |  |
| D13_27 | 2.58 | Bacteroidetes | Porphyromonadaceae | *Porphyromonas* | *Porphyromonas somerae* |
| D13_24 | 2.78 |  |  |  |  |
| D13_20 | 2.94 |  |  |  |  |
| D13_20 | -3.45 | Firmicutes | Enterococcaceae | *Enterococcus* | *Enterococcus sp.* |
| D13_27 | -2.46 |  |  |  |  |
| D13_24 | -2.37 |  |  |  |  |
| D13_20 | 2.69 | Firmicutes | Lactobacillaceae | *Lactobacillus* | *Lactobacillus acidophilus* |
| D13_27 | 2.7 |  |  |  |  |
| D13_24 | 4.25 |  |  |  |  |
| D13_20 | 2.49 | Firmicutes | Lactobacillaceae | *Lactobacillus* | *Lactobacillus crispatus* |
| D13_24 | 2.87 |  |  |  |  |
| D13_27 | 3.24 |  |  |  |  |
| D13_27 | 5.2 | Firmicutes | Lactobacillaceae | *Lactobacillus* | *Lactobacillus delbrueckii* |
| D13_20 | 5.37 |  |  |  |  |
| D13_24 | 6.16 |  |  |  |  |
| D13_24 | 6.76 | Firmicutes | Lactobacillaceae | *Lactobacillus* | *Lactobacillus equicursoris* |
| D13_27 | 7.24 |  |  |  |  |
| D13_20 | 7.55 |  |  |  |  |
| D13_20 | 3.28 | Firmicutes | Lactobacillaceae | *Lactobacillus* | *Lactobacillus gallinarum* |
| D13_24 | 3.93 |  |  |  |  |
| D13_27 | 4.7 |  |  |  |  |
| D13_20 | 2.67 | Firmicutes | Lactobacillaceae | *Lactobacillus* | *Lactobacillus johnsonii* |
| D13_24 | 2.91 |  |  |  |  |
| D13_27 | 3.14 |  |  |  |  |
| D13_27 | 6.97 | Firmicutes | Lactobacillaceae | *Lactobacillus* | *Lactobacillus mucosae* |
| D13_24 | 6.98 |  |  |  |  |
| D13_20 | 7.31 |  |  |  |  |
| D13_20 | 2.94 | Firmicutes | Lactobacillaceae | *Sharpea* | *Sharpea azabuensis* |
| D13_24 | 3.26 |  |  |  |  |
| D13_27 | 3.43 |  |  |  |  |
| D13_24 | 3.79 | Firmicutes | Streptococcaceae | *Streptococcus* | *Streptococcus sp.* |
| D13_20 | 4.09 |  |  |  |  |
| D13_27 | 5.55 |  |  |  |  |
| D13_24 | 2.42 | Firmicutes | Clostridiaceae | *Massilioclostridium* | *Massilioclostridium coli* |
| D13_27 | 2.48 |  |  |  |  |
| D13_20 | 2.51 |  |  |  |  |
| D13_20 | 3.84 | Firmicutes | Eubacteriaceae | *Eubacterium* | *Eubacterium sp.* |
| D13_24 | 4.57 |  |  |  |  |
| D13_27 | 5.22 |  |  |  |  |
| D13_20 | 2.54 | Firmicutes | Lachnospiraceae |  | Lachnospiraceae bacterium |
| D13_24 | 3.69 |  |  |  |  |
| D13_27 | 3.98 |  |  |  |  |
| D13_20 | 2.21 | Firmicutes | Lachnospiraceae | *Blautia* | *Blautia hydrogenotrophica* |
| D13_24 | 3.04 |  |  |  |  |
| D13_27 | 3.56 |  |  |  |  |
| D13_20 | 2.1 | Firmicutes | Lachnospiraceae | *Blautia* | *Blautia sp.* |
| D13_27 | 2.27 |  |  |  |  |
| D13_24 | 2.37 |  |  |  |  |
| D13_20 | 2.05 | Firmicutes | Lachnospiraceae | *Catonella* | *Catonella morbi* |
| D13_24 | 2.56 |  |  |  |  |
| D13_27 | 2.6 |  |  |  |  |
| D13_24 | 2.16 | Firmicutes | Ruminococcaceae | *Gemmiger* | *Gemmiger formicilis* |
| D13_27 | 2.16 |  |  |  |  |
| D13_20 | 2.34 |  |  |  |  |
| D13_20 | 3.3 | Firmicutes | Erysipelotrichaceae |  | Erysipelotrichaceae bacterium |
| D13_27 | 3.37 |  |  |  |  |
| D13_24 | 3.69 |  |  |  |  |
| D13_20 | 2.03 | Firmicutes | Erysipelotrichaceae | *Coprobacillus* | *Coprobacillus sp.* |
| D13_24 | 2.22 |  |  |  |  |
| D13_27 | 3.49 |  |  |  |  |
| D13_24 | 5.31 | Firmicutes | Erysipelotrichaceae | *Dubosiella* | *Dubosiella newyorkensis* |
| D13_20 | 6.54 |  |  |  |  |
| D13_27 | 6.98 |  |  |  |  |
| D13_20 | 3 | Firmicutes | Erysipelotrichaceae | *Holdemanella* | *Holdemanella biformis* |
| D13_27 | 3.01 |  |  |  |  |
| D13_24 | 3.38 |  |  |  |  |
| D13_27 | 2.92 | Firmicutes | Erysipelotrichaceae | *Solobacterium* | *Solobacterium moorei* |
| D13_24 | 3.01 |  |  |  |  |
| D13_20 | 3.41 |  |  |  |  |
| D13_24 | 4.49 | Firmicutes | Veillonellaceae | *Dialister* | *Dialister succinatiphilus* |
| D13_20 | 4.88 |  |  |  |  |
| D13_27 | 5.16 |  |  |  |  |
| D13_27 | 3.84 | Firmicutes | Veillonellaceae | *Megasphaera* | *Megasphaera elsdenii* |
| D13_24 | 4.48 |  |  |  |  |
| D13_20 | 4.76 |  |  |  |  |
| D13_24 | -2.4 | Fusobacteria | Fusobacteriaceae | *Ilyobacter* | *Ilyobacter polytropus* |
| D13_27 | -2.13 |  |  |  |  |
| D13_20 | -2.07 |  |  |  |  |
| D13_24 | 2.31 | Proteobacteria | Helicobacteraceae | *Helicobacter* | *Helicobacter cinaedi* |
| D13_20 | 3.2 |  |  |  |  |
| D13_27 | 4.34 |  |  |  |  |

^*^log2 = log 2 fold change compared to baseline (D13).

**Supplementary Table 4.** Bacterial species (from shotgun data) altered in dogs fed the protein-rich canned diet on days (D) 16, 20, 24, and 27

| Days | log2* | Phylum | Family | Genus | Species |
| --- | --- | --- | --- | --- | --- |
| D13_27 | -5.71 | Actinobacteria | Bifidobacteriaceae | *Bifidobacterium* | *Bifidobacterium animalis* |
| D13_20 | -4.72 |  |  |  |  |
| D13_24 | -4.44 |  |  |  |  |
| D13_16 | -3.42 |  |  |  |  |
| D13_27 | -3.66 | Actinobacteria | Bifidobacteriaceae | *Bifidobacterium* | *Bifidobacterium pseudolongum* |
| D13_20 | -3.5 |  |  |  |  |
| D13_24 | -3.36 |  |  |  |  |
| D13_16 | -2.81 |  |  |  |  |
| D13_27 | -7.2 | Firmicutes | Enterococcaceae |  |  |
| D13_20 | -5.77 |  |  |  |  |
| D13_24 | -5.55 |  |  |  |  |
| D13_16 | -3.79 |  |  |  |  |
| D13_24 | -5.36 | Firmicutes | Streptococcaceae | *Streptococcus* | *Streptococcus dysgalactiae* |
| D13_27 | -5.09 |  |  |  |  |
| D13_20 | -5.05 |  |  |  |  |
| D13_16 | -4.4 |  |  |  |  |
| D13_24 | -5.23 | Firmicutes | Streptococcaceae | *Streptococcus* | *Streptococcus gordonii* |
| D13_20 | -5.18 |  |  |  |  |
| D13_27 | -4.97 |  |  |  |  |
| D13_16 | -4.97 |  |  |  |  |
| D13_27 | -4.82 | Firmicutes | Streptococcaceae | *Streptococcus* | *Streptococcus henryi* |
| D13_24 | -4.61 |  |  |  |  |
| D13_20 | -4.55 |  |  |  |  |
| D13_16 | -3.44 |  |  |  |  |
| D13_20 | -4.97 | Firmicutes | Streptococcaceae | *Streptococcus* | *Streptococcus iniae* |
| D13_24 | -4.78 |  |  |  |  |
| D13_16 | -4.78 |  |  |  |  |
| D13_27 | -4.07 |  |  |  |  |
| D13_27 | -6.25 | Firmicutes | Streptococcaceae | *Streptococcus* | *Streptococcus sp.* |
| D13_20 | -6.03 |  |  |  |  |
| D13_24 | -5.64 |  |  |  |  |
| D13_16 | -4.2 |  |  |  |  |
| D13_27 | -6.61 | Firmicutes | Clostridiaceae | *Clostridium* | *Clostridium mediterraneense* |
| D13_16 | -6.2 |  |  |  |  |
| D13_24 | -5.91 |  |  |  |  |
| D13_20 | -5.36 |  |  |  |  |
| D13_20 | -3.4 | Firmicutes | Erysipelotrichaceae | *Erysipelatoclostridium* | *[Clostridium] spiroforme* |
| D13_16 | -2.93 |  |  |  |  |
| D13_27 | -2.27 |  |  |  |  |
| D13_24 | -2.19 |  |  |  |  |
| D13_16 | 4.07 | Firmicutes | Lachnospiraceae | *Dorea* | *Candidatus Dorea massiliensis* |
| D13_20 | 4.73 |  |  |  |  |
| D13_27 | 5.02 |  |  |  |  |
| D13_24 | 5.24 |  |  |  |  |
| D13_16 | 3.62 | Firmicutes | Lachnospiraceae | *Stomatobaculum* | *Stomatobaculum longum* |
| D13_20 | 3.92 |  |  |  |  |
| D13_24 | 4.27 |  |  |  |  |
| D13_27 | 4.28 |  |  |  |  |
| D13_16 | 5.49 | Firmicutes | Peptostreptococcaceae | *Paeniclostridium* | *Paeniclostridium sordellii* |
| D13_20 | 6.22 |  |  |  |  |
| D13_27 | 6.44 |  |  |  |  |
| D13_24 | 7.09 |  |  |  |  |

^*^log2 = log 2 fold change compared to baseline (D13).

**Supplementary Table 5.** Bacterial species (from shotgun data) altered in dogs fed the protein-rich canned diet on days (D) 16, 20, and 24; and 24 and 27

| Days | log2* | Phylum | Family | Genus | Species |
| --- | --- | --- | --- | --- | --- |
| D13_20 | -2.62 | Firmicutes | Streptococcaceae | *Streptococcus* | *Streptococcus parasanguinis* |
| D13_16 | -2.61 |  |  |  |  |
| D13_24 | -2.1 |  |  |  |  |
| D13_27 | -3.27 | Actinobacteria | Atopobiaceae | *Olsenella* | *Olsenella sp.* |
| D13_24 | -3.25 |  |  |  |  |
| D13_27 | -5.29 | Firmicutes | Enterococcaceae | *Enterococcus* | *Enterococcus columbae* |
| D13_24 | -5.08 |  |  |  |  |
| D13_27 | -3.21 | Firmicutes | Lactobacillaceae | *Lactobacillus* | *Lactobacillus acidophilus* |
| D13_24 | -2.2 |  |  |  |  |
| D13_27 | 4.24 | Firmicutes | Streptococcaceae | *Lactococcus* | *Lactococcus lactis* |
| D13_24 | 5.12 |  |  |  |  |
| D13_27 | -2.7 | Firmicutes | Streptococcaceae | *Streptococcus* | *Streptococcus mitis* |
| D13_24 | -2.57 |  |  |  |  |
| D13_27 | 3.47 | Firmicutes | Lachnospiraceae | *Niameybacter* | *Niameybacter massiliensis* |
| D13_24 | 4.39 |  |  |  |  |
| D13_27 | 2.78 | Firmicutes | Peptostreptococcaceae | *Terrisporobacter* | *Terrisporobacter glycolicus* |
| D13_24 | 2.92 |  |  |  |  |
| D13_24 | -2.61 | Firmicutes | Ruminococcaceae | *Fournierella* | *Fournierella massiliensis* |
| D13_27 | -2.08 |  |  |  |  |
| D13_24 | 2.75 | Proteobacteria | Enterobacteriaceae | *Citrobacter* |  |
| D13_27 | 3.97 |  |  |  |  |

^*^log2 = log 2 fold change compared to baseline (D13).

**Supplementary Table 6.** Bacterial species (from shotgun data) altered in dogs fed the protein-rich canned diet on days (D) 20, 24, and 27

| Days | log2* | Phylum | Family | Genus | Species |
| --- | --- | --- | --- | --- | --- |
| D13_27 | -5.24 | Firmicutes | Lactobacillaceae |  |  |
| D13_24 | -4.53 |  |  |  |  |
| D13_20 | -4.45 |  |  |  |  |
| D13_27 | -5.34 | Firmicutes | Carnobacteriaceae | *Atopostipes* | *Atopostipes suicloacalis* |
| D13_20 | -5.31 |  |  |  |  |
| D13_24 | -4.28 |  |  |  |  |
| D13_27 | -5.99 | Firmicutes | Enterococcaceae | *Enterococcus* | *Enterococcus cecorum* |
| D13_24 | -5.60 |  |  |  |  |
| D13_20 | -5.52 |  |  |  |  |
| D13_24 | -5.22 | Firmicutes | Enterococcaceae | *Enterococcus* | *Enterococcus hermanniensis* |
| D13_27 | -4.57 |  |  |  |  |
| D13_20 | -3.94 |  |  |  |  |
| D13_27 | -5.20 | Firmicutes | Lactobacillaceae | *Lactobacillus* | *Lactobacillus animalis* |
| D13_20 | -4.53 |  |  |  |  |
| D13_24 | -4.52 |  |  |  |  |
| D13_27 | -5.19 | Firmicutes | Lactobacillaceae | *Lactobacillus* | *Lactobacillus murinus* |
| D13_20 | -4.24 |  |  |  |  |
| D13_24 | -3.88 |  |  |  |  |
| D13_27 | -2.83 | Firmicutes | Lactobacillaceae | *Lactobacillus* | *Lactobacillus reuteri* |
| D13_24 | -2.72 |  |  |  |  |
| D13_20 | -2.29 |  |  |  |  |
| D13_27 | -3.59 | Firmicutes | Lactobacillaceae | *Lactobacillus* | *Lactobacillus salivarius* |
| D13_24 | -2.85 |  |  |  |  |
| D13_20 | -2.80 |  |  |  |  |
| D13_27 | -3.68 | Firmicutes | Streptococcaceae | *Streptococcus* | *Streptococcus equinus* |
| D13_20 | -3.03 |  |  |  |  |
| D13_24 | -2.80 |  |  |  |  |
| D13_27 | -3.57 | Firmicutes | Streptococcaceae | *Streptococcus* | *Streptococcus gallolyticus* |
| D13_24 | -3.38 |  |  |  |  |
| D13_20 | -2.47 |  |  |  |  |
| D13_27 | 2.85 | Firmicutes | Streptococcaceae | *Lactococcus* |  |
| D13_20 | 3.13 |  |  |  |  |
| D13_24 | 6.36 |  |  |  |  |
| D13_27 | 2.60 | Firmicutes | Enterococcaceae | *Enterococcus* | *Enterococcus sp.* |
| D13_20 | 2.63 |  |  |  |  |
| D13_24 | 2.90 |  |  |  |  |
| D13_27 | 2.53 | Firmicutes | Streptococcaceae | *Lactococcus* | *Lactococcus garvieae* |
| D13_20 | 2.78 |  |  |  |  |
| D13_24 | 6.00 |  |  |  |  |
| D13_27 | 2.73 | Firmicutes | Streptococcaceae | *Lactococcus* | *Lactococcus sp.* |
| D13_20 | 3.06 |  |  |  |  |
| D13_24 | 6.22 |  |  |  |  |
| D13_20 | -3.78 | Bacteroidetes | Tannerellaceae | *Parabacteroides* | *Parabacteroides sp.* |
| D13_27 | -3.78 |  |  |  |  |
| D13_24 | -3.15 |  |  |  |  |
| D13_24 | -2.53 | Proteobacteria |  |  |  |
| D13_20 | -2.21 |  |  |  |  |
| D13_27 | -2.13 |  |  |  |  |
| D13_24 | -4.40 | Proteobacteria |  |  | *Burkholderiales bacterium* |
| D13_20 | -3.59 |  |  |  |  |
| D13_27 | -3.39 |  |  |  |  |
| D13_20 | -3.85 | Proteobacteria | Sutterellaceae | *Parasutterella* | *Parasutterella excrementihominis* |
| D13_24 | -3.79 |  |  |  |  |
| D13_27 | -3.26 |  |  |  |  |
| D13_24 | -3.36 | Firmicutes | Peptostreptococcaceae | *Romboutsia* | *Romboutsia timonensis* |
| D13_27 | -3.03 |  |  |  |  |
| D13_20 | -2.46 |  |  |  |  |
| D13_20 | 3.92 | Firmicutes | Clostridiaceae | *Clostridium* | *Clostridium paraputrificum* |
| D13_24 | 4.32 |  |  |  |  |
| D13_27 | 5.44 |  |  |  |  |
| D13_27 | 3.49 | Firmicutes | Peptostreptococcaceae | *Paraclostridium* | *Paraclostridium bifermentans* |
| D13_20 | 3.58 |  |  |  |  |
| D13_24 | 4.48 |  |  |  |  |
| D13_24 | -2.64 | Proteobacteria | Campylobacteraceae | *Campylobacter* | *Campylobacter fetus* |
| D13_27 | -2.36 |  |  |  |  |
| D13_20 | -2.06 |  |  |  |  |
| D13_27 | -3.39 | Firmicutes | Erysipelotrichaceae | *Turicibacter* |  |
| D13_24 | -2.69 |  |  |  |  |
| D13_20 | -2.19 |  |  |  |  |
| D13_24 | -4.88 | Firmicutes | Erysipelotrichaceae | *Faecalibaculum* | *Faecalibaculum rodentium* |
| D13_20 | -3.78 |  |  |  |  |
| D13_27 | -3.73 |  |  |  |  |
| D13_27 | -3.92 | Firmicutes | Erysipelotrichaceae | *Turicibacter* | *Turicibacter sp.* |
| D13_20 | -3.15 |  |  |  |  |
| D13_24 | -2.24 |  |  |  |  |
| D13_24 | 2.35 | Proteobacteria | Enterobacteriaceae | *Enterobacter* | *Enterobacter mori* |
| D13_27 | 3.33 |  |  |  |  |
| D13_20 | 4.57 |  |  |  |  |
| D13_24 | -3.86 | Firmicutes | Selenomonadaceae | *Megamonas* | *Megamonas funiformis* |
| D13_20 | -2.87 |  |  |  |  |
| D13_27 | -2.36 |  |  |  |  |
| D13_24 | -5.12 | Firmicutes | Selenomonadaceae | *Megamonas* | *Megamonas hypermegale* |
| D13_20 | -3.88 |  |  |  |  |
| D13_27 | -3.37 |  |  |  |  |
| D13_24 | -4.26 | Firmicutes | Selenomonadaceae | *Megamonas* | *Megamonas rupellensis* |
| D13_27 | -3.89 |  |  |  |  |
| D13_20 | -2.98 |  |  |  |  |

^*^log2 = log 2 fold change compared to baseline (D13).

**Supplementary Table 7.** Bacterial phyla and genera (% of total sequences) from 16S rRNA sequencing in feces of dogs fed a high-fiber or protein-rich canned diet on days (D) 13, 16, 20, 24, and 27

| **Phylum** | **Genus** | **High-fiber diet** | | | | | | **Canned diet** | | | | | |  | | **P-value** | | |
| --- | --- | --- | --- | --- | --- | --- | --- | --- | --- | --- | --- | --- | --- | --- | --- | --- | --- | --- |
|  |  | **D13** | **D16** | **D20** | **D24** | **D27** | **D13** | | **D16** | **D20** | **D24** | **D27** | **SEM** | | **Diet** | | **Time** | **Diet*Time** |
| Actinobacteria |  | 0.27 | 0.41 | 0.99* | 0.74 | 0.72 | 0.48 | | 0.16 | 0.18 | 0.21 | 0.18 | 0.60 | | <0.0001 | | 0.2150 | 0.0029 |
|  | *Bifidobacterium* | 0.03 | 0.00 | 0.62* | 0.19 | 0.24 | 0.16 | | 0.02 | 0.00 | 0.00 | 0.00 | 0.32 | | 0.0071 | | 0.0574 | 0.0055 |
| Bacteroidetes |  | 25.54 | 29.88 | 30.70 | 28.52 | 30.72 | 26.69 | | 28.46 | 30.60 | 27.85 | 27.55 | 4.89 | | 0.3465 | | 0.0302 | 0.6307 |
|  | *Bacteroides* | 15.80 | 21.61 | 19.92 | 14.45* | 16.18 | 16.86 | | 19.82 | 23.29 | 21.10 | 21.24 | 3.98 | | 0.0016 | | 0.0016 | 0.0288 |
|  | *Parabacteroides* | 0.39 | 0.91 | 0.99 | 1.80* | 1.71* | 0.33 | | 0.50 | 0.19 | 0.18 | 0.23 | 0.78 | | <0.0001 | | 0.0574 | 0.0065 |
|  | *Prevotella* | 0.84 | 0.79 | 2.83* | 4.24* | 6.09* | 0.69 | | 0.30 | 0.18 | 0.11 | 0.19 | 1.16 | | <0.0001 | | <0.0001 | <0.0001 |
| Firmicutes |  | 26.33 | 30.41* | 36.07* | 41.00* | 40.88* | 25.21 | | 18.09 | 17.36 | 23.09 | 21.12 | 4.83 | | <0.0001 | | 0.0037 | 0.0006 |
|  | *Allobaculum* | 2.90 | 0.61 | 1.22 | 1.31 | 3.63 | 3.07 | | 0.84 | 0.25 | 0.50 | 0.47 | 1.11 | | 0.0964 | | 0.0318 | 0.2815 |
|  | *Blautia* | 2.65 | 5.05* | 4.08^#^ | 6.33* | 6.36* | 2.54 | | 1.69 | 2.11 | 3.30 | 2.92 | 1.76 | | <0.0001 | | <0.0001 | 0.0018 |
|  | *Catenibacterium* | 0.01 | 0.37 | 0.74 | 1.44* | 1.59* | 0.01 | | 0.01 | 0.01 | 0.01 | 0.02 | 0.59 | | <0.0001 | | 0.0003 | 0.0004 |
|  | *Clostridium* | 6.31 | 5.52 | 4.78 | 4.91^#^ | 4.00* | 7.31 | | 7.25 | 6.76 | 8.74 | 8.52 | 2.31 | | <0.0001 | | 0.7378 | 0.2302 |
|  | *Coprobacillus* | 0.10 | 0.11* | 0.02 | 0.02 | 0.05 | 0.08 | | 0.02 | 0.03 | 0.05 | 0.05 | 0.21 | | 0.2620 | | 0.0081 | 0.0227 |
|  | *Dorea* | 1.43 | 0.53 | 0.26 | 0.38 | 0.31 | 1.24 | | 0.53 | 0.65 | 0.80 | 0.63 | 0.75 | | 0.0382 | | <0.0001 | 0.1289 |
|  | *Faecalibacterium* | 1.57 | 1.20 | 1.76* | 1.49^#^ | 2.00* | 1.40 | | 0.67 | 0.51 | 0.50 | 0.66 | 0.99 | | <0.0001 | | 0.0548 | 0.0309 |
|  | *Lactobacillus* | 1.37 | 2.72 | 5.41* | 4.18 | 1.66 | 0.36 | | 0.10 | 0.00 | 0.00 | 0.00 | 1.15 | | <0.0001 | | 0.2515 | 0.1572 |
|  | *Megamonas* | 1.01 | 3.18* | 2.74* | 2.62* | 2.91* | 1.30 | | 0.92 | 0.10 | 0.05 | 0.09 | 1.12 | | <0.0001 | | 0.1952 | 0.0004 |
|  | *Phascolarctobacterium* | 1.14 | 0.90 | 0.97 | 1.09 | 1.19 | 1.21 | | 0.70 | 0.72 | 0.67 | 0.75 | 0.88 | | 0.0018 | | 0.0245 | 0.2487 |
|  | *Ruminococcus* | 0.02 | 0.03 | 0.03 | 0.04 | 0.04* | 0.01 | | 0.02 | 0.02 | 0.02 | 0.01 | 0.14 | | <0.0001 | | 0.0045 | 0.2386 |
|  | *Streptococcus* | 1.40 | 1.53 | 1.07 | 2.64^#^ | 2.12 | 0.82 | | 0.04 | 0.04 | 0.08 | 0.01 | 0.90 | | <0.0001 | | 0.6791 | 0.4382 |
| Fusobacteria |  | 36.98 | 26.31* | 19.43* | 18.40* | 16.25* | 33.09 | | 35.09 | 35.72 | 32.75 | 36.00 | 4.92 | | <0.0001 | | <0.0001 | <0.0001 |
|  | *Fusobacterium* | 12.08 | 11.26* | 9.06 | 9.15 | 7.12* | 11.93 | | 15.06 | 16.45 | 13.10 | 13.99 | 3.15 | | <0.0001 | | 0.1558 | 0.0148 |
| Proteobacteria |  | 10.79 | 12.82^#^ | 12.67 | 11.17 | 11.30 | 14.49 | | 18.08 | 15.91 | 15.95 | 14.97 | 3.39 | | <0.0001 | | 0.1605 | 0.9084 |
|  | *Anaerobiospirillum* | 1.47 | 5.24 | 5.05 | 4.57 | 3.60 | 3.45 | | 3.11 | 1.28 | 1.56 | 1.72 | 1.61 | | 0.0400 | | 0.3691 | 0.0211 |
|  | *Sutterella* | 8.73 | 6.85* | 7.04* | 6.10* | 6.90* | 8.65 | | 14.48 | 13.40 | 13.70 | 12.59 | 2.86 | | 0.0038 | | 0.4115 | 0.0295 |

*Means values within a day were different between diets.

^#^Means values within a day tended to be different between diets.

**Supplementary Table 8.** Bacterial gene (KO term) abundance that increased in feces of dogs fed the high-fiber diet

| log2* | KO term | Day | Metabolite |
| --- | --- | --- | --- |
| 3.80 | K00002 | D13vsD16 | alcohol dehydrogenase (NADP+) |
| 3.90 | K00002 | D13vsD20 |  |
| 3.90 | K00002 | D13vsD27 |  |
| 4.42 | K00002 | D13vsD24 |  |
| 2.28 | K00282 | D13vsD20 | glycine dehydrogenase subunit 1 |
| 2.47 | K00282 | D13vsD27 |  |
| 2.48 | K00282 | D13vsD24 |  |
| 2.89 | K00282 | D13vsD16 |  |
| 2.14 | K00283 | D13vsD20 | glycine dehydrogenase subunit 2 |
| 2.24 | K00283 | D13vsD24 |  |
| 2.52 | K00283 | D13vsD27 |  |
| 2.53 | K00283 | D13vsD16 |  |
| 2.51 | K00805 | D13vsD24 | heptaprenyl diphosphate synthase component 1 |
| 2.62 | K00805 | D13vsD16 |  |
| 2.67 | K00805 | D13vsD20 |  |
| 2.82 | K00805 | D13vsD27 |  |
| 2.91 | K01060 | D13vsD20 | cephalosporin-C deacetylase |
| 2.96 | K01060 | D13vsD27 |  |
| 3.16 | K01060 | D13vsD16 |  |
| 3.57 | K01060 | D13vsD24 |  |
| 2.03 | K19548 | D13vsD16 | dihydroanticapsin dehydrogenase |
| 2.16 | K19548 | D13vsD24 |  |
| 2.21 | K19548 | D13vsD27 |  |
| 2.23 | K19548 | D13vsD20 |  |
| 3.04 | K08681 | D13vsD16 | pyridoxal 5'-phosphate synthase pdxT subunit |
| 3.33 | K08681 | D13vsD20 |  |
| 3.34 | K08681 | D13vsD27 |  |
| 3.49 | K08681 | D13vsD24 |  |
| 2.97 | K11782 | D13vsD20 | chorismate dehydratase |
| 2.99 | K11782 | D13vsD24 |  |
| 3.11 | K11782 | D13vsD16 |  |
| 3.39 | K11782 | D13vsD27 |  |
| 2.98 | K18285 | D13vsD20 | aminodeoxyfutalosine synthase |
| 3.11 | K18285 | D13vsD24 |  |
| 3.29 | K18285 | D13vsD16 |  |
| 3.40 | K18285 | D13vsD27 |  |
| 3.22 | K13919 | D13vsD20 | propanediol dehydratase medium subunit |
| 3.26 | K13919 | D13vsD16 |  |
| 3.39 | K13919 | D13vsD27 |  |
| 3.74 | K13919 | D13vsD24 |  |
| 3.31 | K13920 | D13vsD16 | propanediol dehydratase small subunit |
| 3.37 | K13920 | D13vsD20 |  |
| 3.38 | K13920 | D13vsD27 |  |
| 3.84 | K13920 | D13vsD24 |  |
| 2.71 | K18284 | D13vsD16 | adenosylhomocysteineaminodeoxyfutalosine nucleosidase |
| 2.98 | K18284 | D13vsD20 |  |
| 3.20 | K18284 | D13vsD27 |  |
| 3.39 | K18284 | D13vsD24 |  |
| 3.75 | K00619 | D13vsD20 | amino-acid N-acetyltransferase |
| 4.00 | K00619 | D13vsD16 |  |
| 4.14 | K00619 | D13vsD24 |  |
| 4.51 | K00619 | D13vsD27 |  |
| 2.75 | K01251 | D13vsD20 | adenosylhomocysteinase |
| 2.85 | K01251 | D13vsD24 |  |
| 2.96 | K01251 | D13vsD27 |  |
| 3.03 | K01251 | D13vsD16 |  |
| 2.56 | K11784 | D13vsD20 | cyclic dehypoxanthinyl futalosine synthase |
| 2.82 | K11784 | D13vsD24 |  |
| 3.25 | K11784 | D13vsD27 |  |
| 2.88 | K12252 | D13vsD20 | arginine:pyruvate transaminase |
| 3.15 | K12252 | D13vsD27 |  |
| 3.71 | K12252 | D13vsD24 |  |
| 4.08 | K14369 | D13vsD20 | erythromycin 3''-O-methyltransferase |
| 4.10 | K14369 | D13vsD27 |  |
| 4.45 | K14369 | D13vsD24 |  |
| 2.81 | K18429 | D13vsD20 | GDP/UDP-N,N'-diacetylbacillosamine 2-epimerase |
| 3.18 | K18429 | D13vsD27 |  |
| 3.68 | K18429 | D13vsD24 |  |
| 2.52 | K02788 | D13vsD20 | lactose PTS system EIICB component |
| 3.21 | K02788 | D13vsD27 |  |
| 3.44 | K02788 | D13vsD24 |  |
| 2.32 | K00824 | D13vsD24 | D-alanine transaminase |
| 2.75 | K00824 | D13vsD27 |  |
| 3.06 | K15895 | D13vsD24 | UDP-4-amino-4,6-dideoxy-L-N-acetyl-beta-L-altrosamine transaminase |
| 3.25 | K15895 | D13vsD27 |  |
| 3.84 | K16967 | D13vsD24 | dimethyl-sulfide monooxygenase |
| 4.08 | K16967 | D13vsD27 |  |
| 3.80 | K05299 | D13vsD24 | formate dehydrogenase (NADP+) alpha subunit |
| 3.90 | K05299 | D13vsD27 |  |

^*^log2 = log 2 fold change compared to baseline (D13).

**Supplementary Table 9.** Bacterial gene (KO term) abundance that decreased in feces of dogs fed the high-fiber diet

| log2* | KO term | Day | Metabolite |
| --- | --- | --- | --- |
| -2.43 | K20625 | D13vsD24 | acetylene hydratase |
| -2.41 | K20625 | D13vsD16 |  |
| -2.00 | K20625 | D13vsD27 |  |
| -3.96 | K01039 | D13vsD27 | glutaconate CoA-transferase, subunit A |
| -3.49 | K01039 | D13vsD20 |  |
| -2.51 | K01039 | D13vsD24 |  |
| -3.00 | K05350 | D13vsD20 | beta-glucosidase |
| -2.90 | K05350 | D13vsD27 |  |
| -2.44 | K05350 | D13vsD24 |  |
| -3.95 | K18067 | D13vsD20 | phthalate 4,5-cis-dihydrodiol dehydrogenase |
| -3.91 | K18067 | D13vsD27 |  |
| -3.52 | K18067 | D13vsD24 |  |
| -2.59 | K12234 | D13vsD27 | coenzyme F420-0:L-glutamate ligase / coenzyme F420-1:gamma-L-glutamate ligase |
| -2.47 | K12234 | D13vsD24 |  |
| -2.31 | K17717 | D13vsD24 | phospholipase D |
| -2.12 | K17717 | D13vsD27 |  |
| -4.05 | K18770 | D13vsD27 | penicillin-binding protein 4 |
| -3.80 | K18770 | D13vsD24 |  |
| -2.33 | K20573 | D13vsD24 | 2'-deamino-2'-hydroxyneamine 1-alpha-D-kanosaminyltransferase |
| -2.32 | K20573 | D13vsD27 |  |

^*^log2 = log 2 fold change compared to baseline (D13).

**Supplementary Table 10.** Bacterial gene (KO term) abundance that were altered in feces of dogs fed the protein-rich canned diet

| log2* | KO term | Days | Metabolite |
| --- | --- | --- | --- |
| 3.27 | K00367 | D13vsD20 | ferredoxin-nitrate reductase |
| 3.63 | K00367 | D13vsD27 |  |
| 3.84 | K00367 | D13vsD24 |  |
| 2.44 | K13919 | D13vsD27 | propanediol dehydratase medium subunit |
| 2.47 | K13919 | D13vsD20 |  |
| 3.07 | K13919 | D13vsD24 |  |
| 2.15 | K16043 | D13vsD20 | scyllo-inositol 2-dehydrogenase (NAD+) |
| 2.41 | K16043 | D13vsD27 |  |
| 2.65 | K16043 | D13vsD24 |  |
| 3.35 | K17217 | D13vsD20 | cystathionine gamma-lyase / homocysteine desulfhydrase |
| 3.53 | K17217 | D13vsD27 |  |
| 4.11 | K17217 | D13vsD24 |  |
| 3.03 | K02510 | D13vsD24 | 4-hydroxy-2-oxoheptanedioate aldolase |
| 3.89 | K02510 | D13vsD20 |  |
| 3.34 | K00131 | D13vsD27 | glyceraldehyde-3-phosphate dehydrogenase (NADP+) |
| 3.73 | K00131 | D13vsD24 |  |
| 2.03 | K12308 | D13vsD24 |  |
| 2.25 | K03335 | D13vsD27 | inosose dehydratase |
| 2.61 | K03335 | D13vsD24 |  |
| 2.34 | K03338 | D13vsD27 | 5-dehydro-2-deoxygluconokinase |
| 2.58 | K03338 | D13vsD24 |  |
| 3.91 | K03339 | D13vsD27 | 6-phospho-5-dehydro-2-deoxy-D-gluconate aldolase |
| 3.95 | K03339 | D13vsD24 |  |
| 3.5 | K16619 | D13vsD27 | phospholipase C / alpha-toxin |
| 3.87 | K16619 | D13vsD24 |  |
| 3.2 | K10209 | D13vsD27 | 4,4'-diapophytoene desaturase |
| 3.54 | K10209 | D13vsD24 |  |
| 3.38 | K12503 | D13vsD27 | short-chain Z-isoprenyl diphosphate synthase |
| 3.42 | K12503 | D13vsD24 |  |
| 2.96 | K16050 | D13vsD27 | 4,5:9,10-diseco-3-hydroxy-5,9,17-trioxoandrosta-1(10),2-diene-4-oate hydrolase |
| 3.48 | K16050 | D13vsD24 |  |
| 3.05 | K00372 | D13vsD27 | assimilatory nitrate reductase catalytic subunit |
| 3.25 | K00372 | D13vsD24 |  |
| 3.04 | K16950 | D13vsD27 | anaerobic sulfite reductase subunit A |
| 3.41 | K16950 | D13vsD24 |  |
| 2.55 | K16951 | D13vsD27 | anaerobic sulfite reductase subunit B |
| 2.89 | K16951 | D13vsD24 |  |
| 3.43 | K05979 | D13vsD27 | 2-phosphosulfolactate phosphatase |
| 3.92 | K05979 | D13vsD24 |  |
| 3.3 | K01590 | D13vsD27 | histidine decarboxylase |
| 3.66 | K01590 | D13vsD24 |  |
| 2.54 | K11440 | D13vsD27 | choline dehydrogenase |
| 2.87 | K11440 | D13vsD24 |  |
| 3.73 | K17216 | D13vsD24 | cystathionine beta-synthase (O-acetyl-L-serine) |
| 3.84 | K17216 | D13vsD27 |  |
| 2.01 | K02825 | D13vsD27 | pyrimidine operon attenuation protein / uracil phosphoribosyltransferase |
| 2.12 | K02825 | D13vsD24 |  |
| -4.75 | K00689 | D13vsD27 | dextransucrase |
| -4.2 | K00689 | D13vsD24 |  |
| -3.89 | K00689 | D13vsD20 |  |

^*^log2 = log 2 fold change compared to baseline (D13).


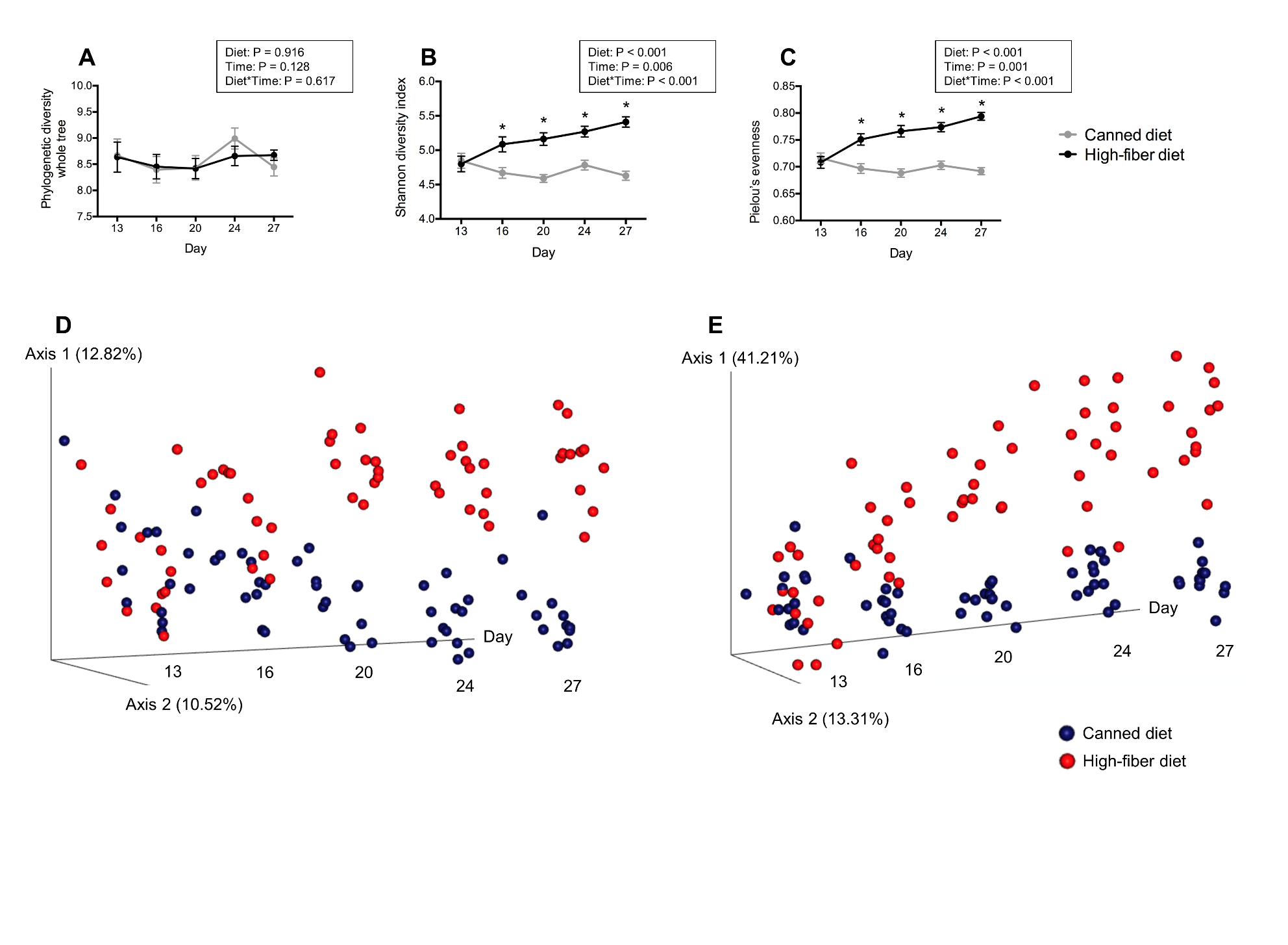


**Supplementary Figure 1.** Fecal microbiota communities of dogs fed a high-fiber diet or protein-rich canned diet from 16S rRNA sequencing. Alpha diversity measures: phylogenetic diversity (A), Shannon diversity index (B), and Pielou’s evenness (C). Beta diversity measures show distinct separation of dogs transitioned to HFD and CD were noted by d 20 for unweighted Unifrac distance (D) and d 16 for weighted Unifrac distance (E). *Mean values within time points were different between diets (P < 0.05); #Mean values within time points tended to be different between diets (P < 0.10).


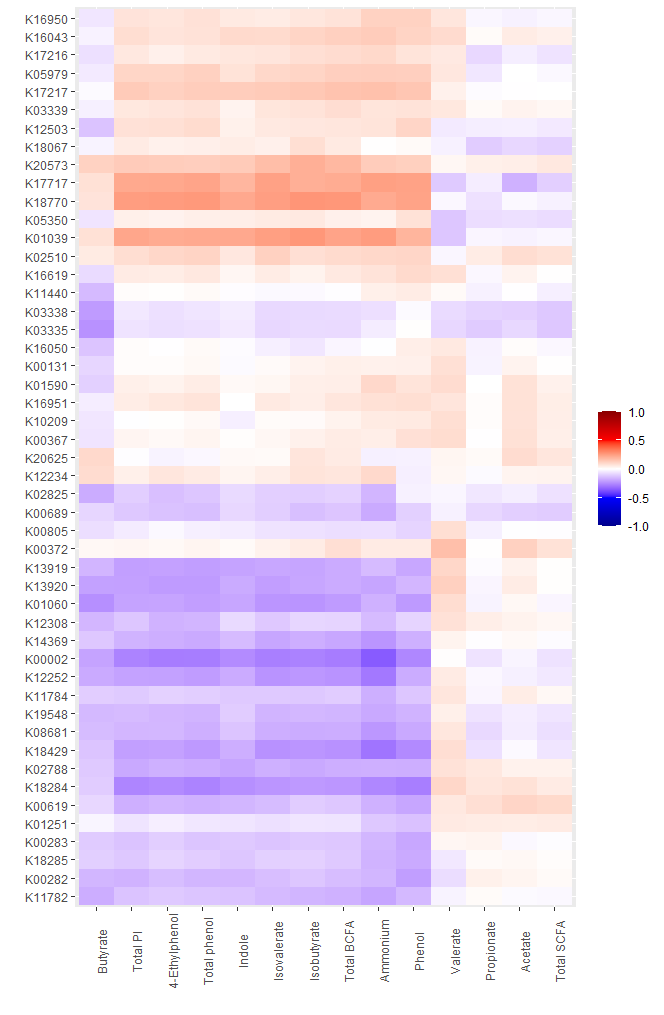


+

**Supplementary Figure 2**. Heatmap of significant correlation values (r) between fecal KO terms and fecal metabolites. The X and Y axes of the thermal graph are the metabolites and KO terms, respectively. R values are represented by different colors (red: positive; blue: negative). Significant correlations (adj P < 0.05) are indicated by ‘ + ’.
